# Development of an open source laboratory information management system for 2-D gel electrophoresis-based proteomics workflow

**DOI:** 10.1186/1471-2105-7-430

**Published:** 2006-10-04

**Authors:** Hiraku Morisawa, Mikako Hirota, Tosifusa Toda

**Affiliations:** 1 Proteomics Collaboration Center, Tokyo Metropolitan Institute of Gerontology, 35-2 Sakaecho, Itabashi-ku, Tokyo, 173-0015, Japan

## Abstract

**Background:**

In the post-genome era, most research scientists working in the field of proteomics are confronted with difficulties in management of large volumes of data, which they are required to keep in formats suitable for subsequent data mining. Therefore, a well-developed open source laboratory information management system (LIMS) should be available for their proteomics research studies.

**Results:**

We developed an open source LIMS appropriately customized for 2-D gel electrophoresis-based proteomics workflow. The main features of its design are compactness, flexibility and connectivity to public databases. It supports the handling of data imported from mass spectrometry software and 2-D gel image analysis software. The LIMS is equipped with the same input interface for 2-D gel information as a clickable map on public 2DPAGE databases. The LIMS allows researchers to follow their own experimental procedures by reviewing the illustrations of 2-D gel maps and well layouts on the digestion plates and MS sample plates.

**Conclusion:**

Our new open source LIMS is now available as a basic model for proteome informatics, and is accessible for further improvement. We hope that many research scientists working in the field of proteomics will evaluate our LIMS and suggest ways in which it can be improved.

## Background

As part of the Human Genome Project that was carried out by an international consortium, a laboratory information management system (LIMS) was developed for genome research and evaluated as an essential tool for advanced studies in the life sciences [1,2]. It is now widely expected that in the post-genome era considerable advances will be made in the ongoing Human Proteome Project. Many research scientists working in the field of proteomics are required to manage huge volumes of experimental data, which are obtained by two-dimensional polyacrylamide gel electrophoresis (2-DPAGE), image analysis, mass spectrometry (MS) analysis and related information downloaded from the public databases in field of life science. Therefore there is a need to develop another proteomics-oriented LIMS in order to manage large volumes of proteomic data more efficiently.

Most of the software for 2-D gel image analysis carries out detection of protein spots on 2-D gel images, matching the spots among multiple gel images and quantifying the spot density automatically. The software for mass spectrometry analysis picks out peaks in mass spectra and searches them against a database, the so-called "peptide-mass fingerprint", for protein identification.

Members of a research project team are often required to carefully consider their experimental schedules, which have to run in parallel with the processing of data imported from various analyzers. A LIMS that is optimized for proteomics would undoubtedly be helpful for scheduling experiments in proteomic research projects and for processing bulk data imported from 2-D gel image analyzers and mass spectrometers. At the same time, it has been widely acknowledged since the inception of proteomics research that data sharing among researchers worldwide is essential. Accordingly, many research groups have attempted to construct public proteome databases on web-based servers.

In many of these proteome databases, information about protein masses, post-translational modifications, expression and variation have been assembled onto 2-D gel images. These are known as "2DPAGE databases", such as SWISS-2DPAGE [3] and TMIG-2DPAGE [4,5]. It is expected that a LIMS for proteomics will adopt the same approach as that of the 2DPAGE database. In 2002, Cho and co-workers developed an original LIMS for proteome research (YPRC-PDB) [6], constructed using a commercial relational database (RDB), Oracle8i. The interface of the database was designed for web browsing with PHP3, and managed data imported from 2-Dgel image analyzers and mass spectrometers. They intended to establish YRC-PDB as a proteome data warehouse. In 2003, Goh and co-workers developed SPINE2, a LIMS for structural proteomics [7], constructed with MySQL and Perl, and also designed to work as a pipeline to public data resources. In 2004, Garwood and co-workers developed PEDRo, the Proteomics Experimental Data Repository [8,9], constructed with a native XML database, Xindice with an ambitious Apache Software Foundation basis. The XML-based document format has better quality for communication than the other formats. The native XML database has great potential, but may have critical limitations for proteomic research. On the other hand, commercially available LIMSes (Amersham Biosciences [10] and Bio-Rad Laboratories Inc, etc.) have also been developed and released, but they are not exactly suitable for most small laboratories like ours.

Many of the LIMS for proteomics designed in the past have a web interface instead of special client software, and have adopted the format of an Internet-based public proteome database. Moreover they have often been linked to public proteome databases, and attempted to support XML format as a communication format.

For researchers in the field of proteomics, it would be highly advantageous to develop a LIMS that would allow export and import of data in a standard format. The Human Proteome Organization Proteomics Standards Initiative, HUPO-PSI, began steps to establish a standard format in 2004 [11-13]. Initially, an attempt was made to standardize the items for data representation and exchange. Steps were then taken to standardize the linkage between the items in XML format for the various workflows of mass spectrometry analysis. Now all of the HUPO-PSI is in XML format, and includes many items and links among them. The XML format is excellent for communication but not so suitable for data management in a relational database system. The appropriate data capacity of a LIMS and optimal performance for data management depend on the total proteomics system used in each laboratory.

We have developed an original open source LIMS for 2-D gel electrophoresis-based proteomics workflow on the basis of the above background. The major features of our LIMS are compactness, flexibility, and connectivity to public databases.

## Implementation

### 1. Software and hardware architecture

We developed the LIMS on the PC Servers PowerEdge 700 and PowerEdge SC420 (Dell Corp.). The operating system is Red Hat Linux 9. We decided to use a web browser as the user interface of the LIMS, because it is universally available on most client systems, even though it is not a full-featured database client. Internet Explorer version 6.0 or later, Netscape version 7.1 or later, or Firefox version 1.03 or later should be installed in the client PC. We also adopted a PHP-Hypertext Preprocessor 4.3.7-involved GD-Graphics Library to make the screen of the web page dynamic. PHP works as an interface between the web server and RDB in our LIMS. Our LIMS has the typical architecture of an "Apache-PHP-PostgreSQL" system. Within the framework of the interfaces of the RDB, although Java is a more portable software that runs well on a variety of computing platforms, we decided to use PHP because it is easier for software programming and has a better performance.

The contents of the RDB include raw data files imported from Kompact mass spectrometry software (Kratos Analytical Ltd.), PDF files (Portable Document Format) from the Mascot database search system (Matrix Science Ltd.), and JPEG image files from the PDQUEST image analyzing system (Bio-Rad Laboratories Inc.). Kompact, Adobe Reader and some other application software packages are needed on the client system for the LIMS. They must be registered as helper applications to work on the web browser.

The inclusion of raw data in the contents makes the LIMS architecture simpler, but disturbs data conversion for exportation. The contents of the RDB also include datasets for constructing our public TMIG-2DPAGE database. We convert the datasets of the LIMS to the contents of the mirror TMIG-2DPAGE database in our institution's intranet. The XML format data and JPEG image files in the TMIG-2DPAGE database are opened for public access via the Internet. While developing the system, many PHP script files became extremely complex, but improvable. Our LIMS is not designed to work only on a very high-performance hardware system. We verified the performance of the system client using Internet Explorer 6.0, Netscape 7.1 and Firefox 1.5 on the Windows XP platform, and using Netscape 7.1 on the MacOS 9.1 and MacOS 10.3 platforms.

### 2. Workflow

To access the LIMS server using a web-based client PC, the user must first login with an authorized username and a given password [see Additional file 2]. At the login page, users are guided to the next layer of data entry in two ways. One is a "menu selection mode", which lists material ID, gel method ID, analysis method ID, gel ID, digestion plate ID, MS plate ID or map ID that correspond to the gel ID in the 2DPAGE database. Small gel images are displayed as icons in the gel ID and the map ID lists. All members of a research team in a laboratory share usernames and all content IDs. Convenience is considered more important than absolute security in our proteome LIMS, which is optimized for small laboratories in universities and academic institutions like ours.

The second guide mode is a "keyword search mode". To access the result of a keyword search, users must enter their username and a given password on the login page. They are then allowed to enter or edit data at any step of the proteomics workflow.

**Figure 1:**
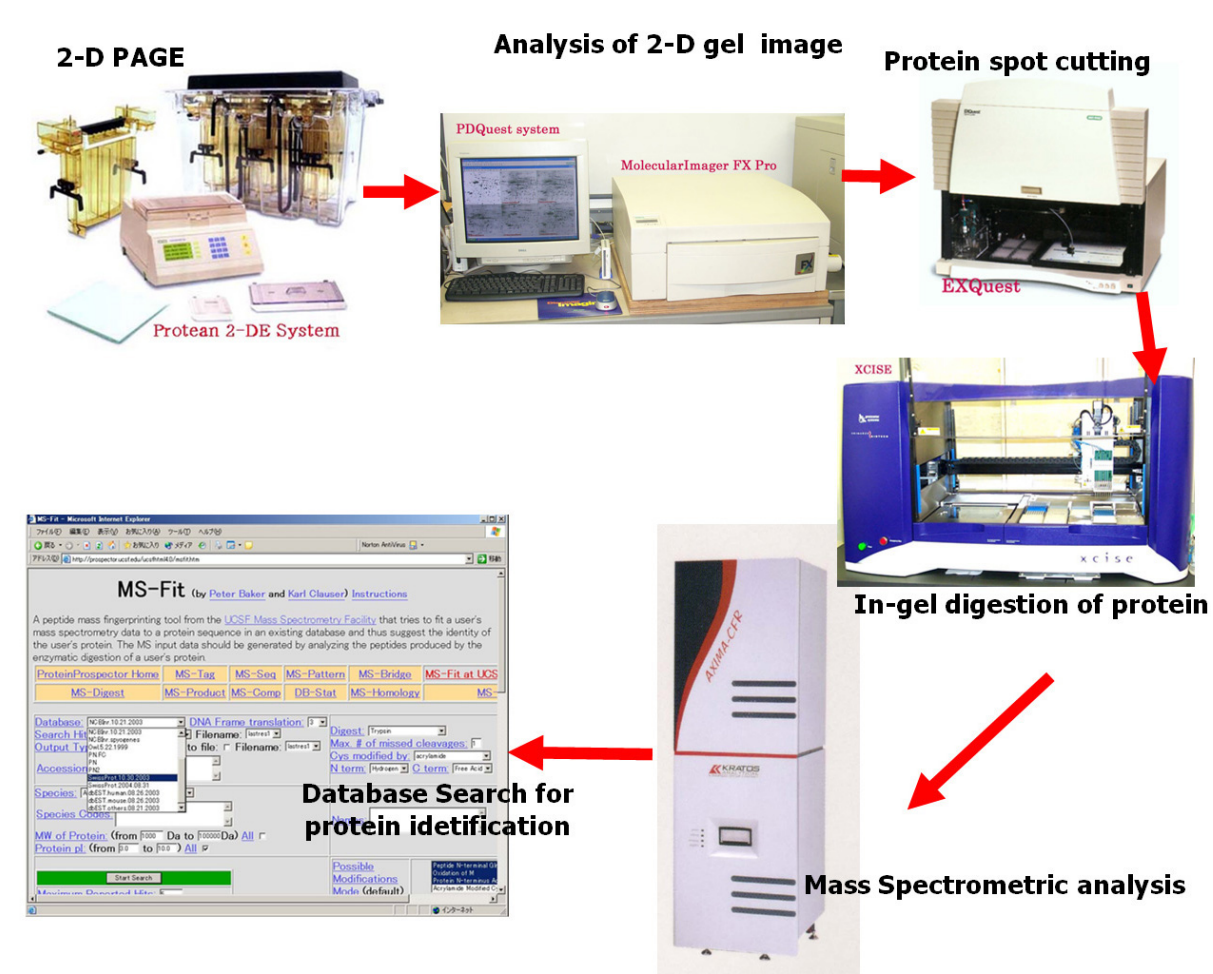
**A general procedure in 2-D PAGE-based proteomics workflow**. After separation of proteins by 2-D PAGE, we acquire 2-D gel images using a laser-scanning imager system and analyze the gel images with the expert software in the 2-D PAGE-based proteomics workflow. And we operate in-gel digestions using the expert machine after the spot cutting of 2-D PAGE gel. We analyze the digested peptide masses and search database for protein identification using the search tool of MS-fit (The Regents of the University of California) [15] or Mascot (Matrix Science Ltd) following Mass Spectrometric analysis using the expert equipment.

**Figure 2:**
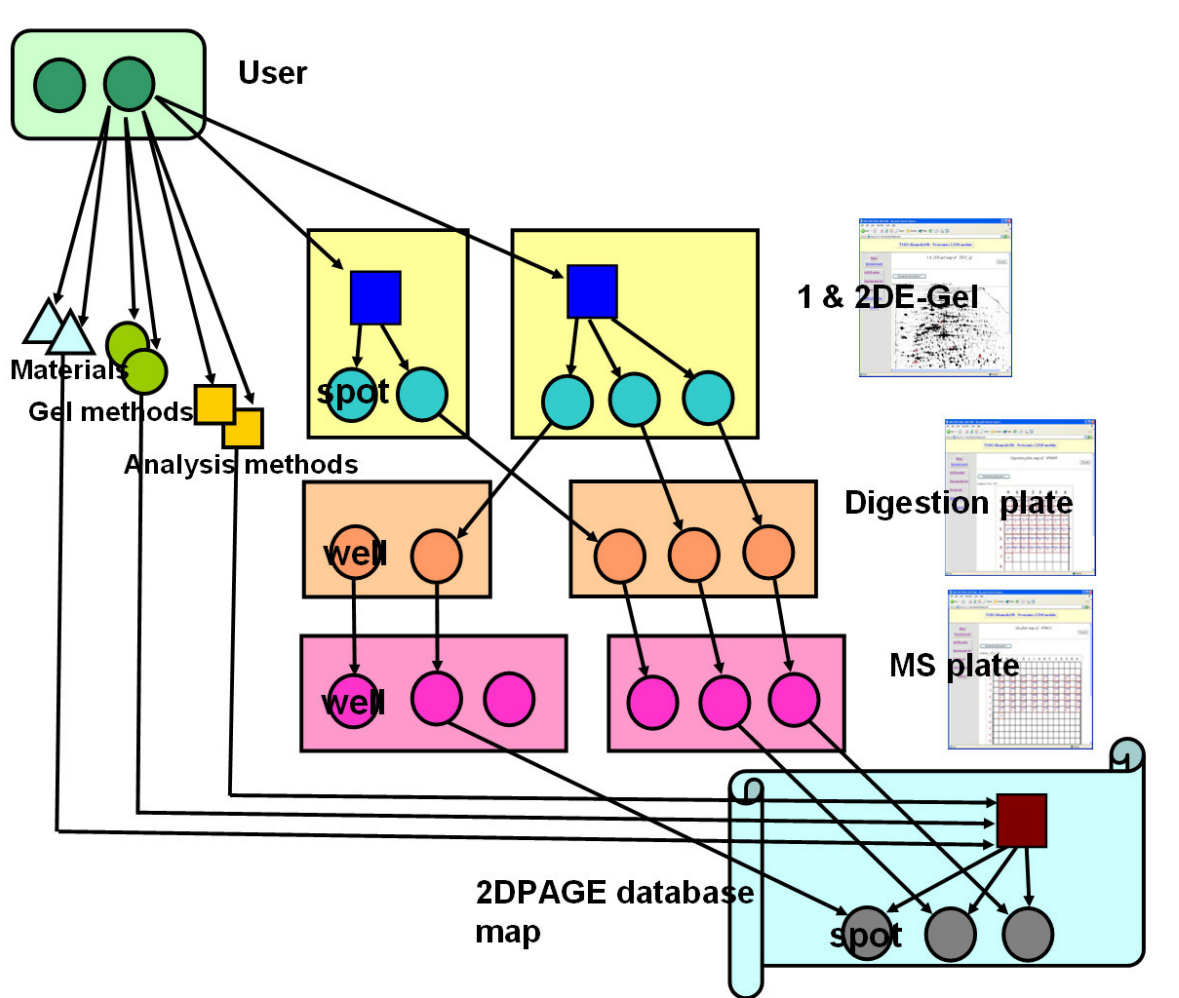
**A scheme of the data flow in the LIMS**. We register and restructure the experimental data following the workflow for entering the data into our LIMS.

Figure 1 shows an example of the basic proteomics workflow process. Figure 2 shows the corresponding data flow process in the LIMS. The first step in the workflow is entering information about materials, gel methods and parameters for operation of analyzers. The next step is entering the image ID of scanned 1-D & 2-D PAGE gels, the standard spot number (SSP number), the locations of spots in the gel image, date and free memo using a clickable function like a public 2DPAGE database, and upload the image files of scanned 1-D & 2-D PAGE gel images. The third step is entering the well ID for the digestion plate, date, free memo and the corresponding SSP number in the gel. The fourth step is entering the well ID for the MS plate, date, free memo, the binary data of mass spectrometry analysis and the corresponding well ID in the digestion plate. The last step is entering the dataset for public access via the Internet and the corresponding well ID in the MS plate.

**Figure 3:**
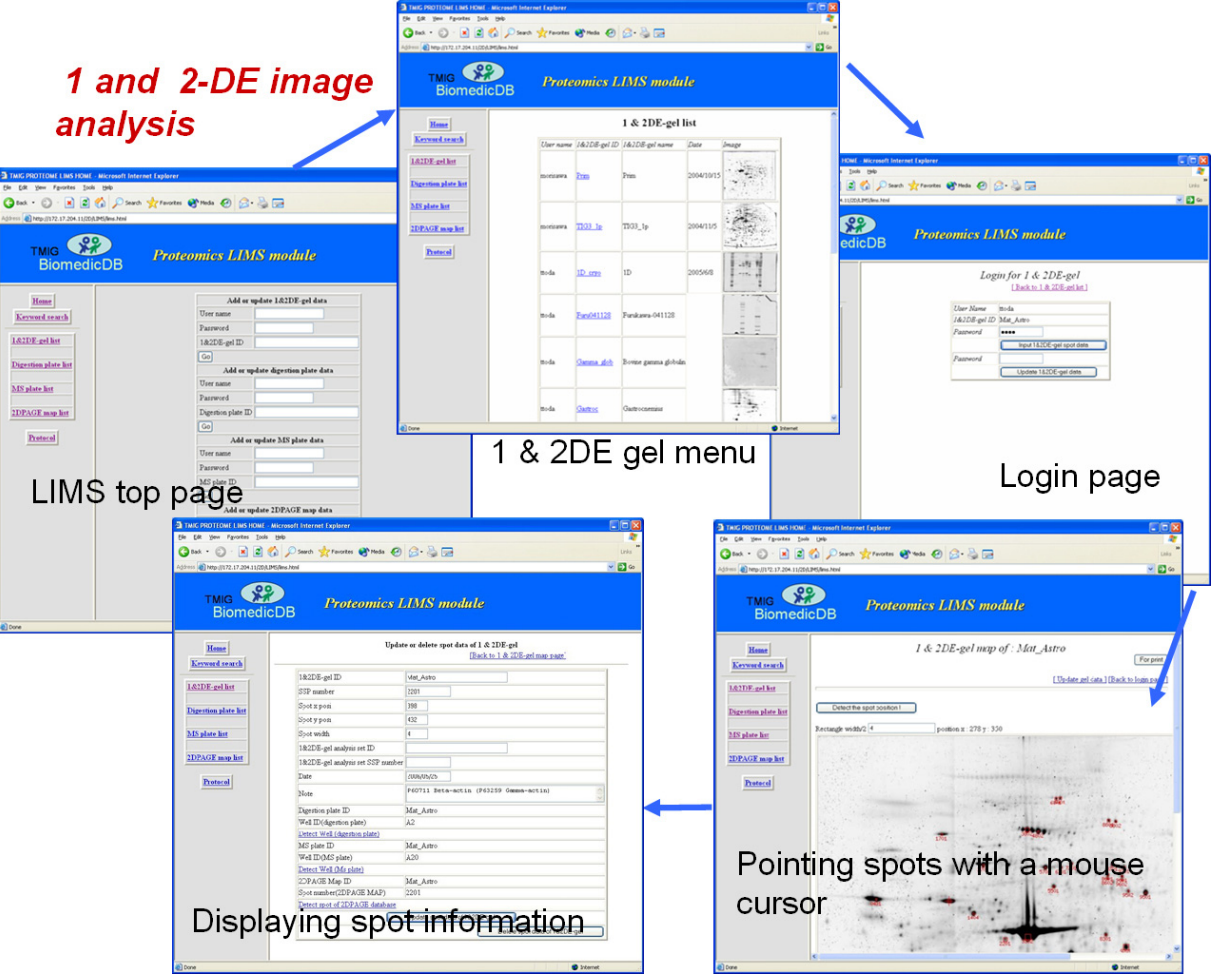
**The graphic user interface for 1 and 2-DE image analyses**. LIMS users are allowed to register new data and operate the experimental data through web interface. They are able to use gel list window with icon images, user login window, gel image window, spot information window and etc for 1 and 2-DE image analyses.

Figure 3 shows the graphical user interface (GUI) flow for entering the data for 1-D & 2-D PAGE image analysis. The windows of Internet Explorer 6.0 in Figure 3 are the top pages of the LIMS, 1-D & 2-D gel menu, and login page, detecting the spot and displaying the spot information. At first, the user must enter the gel information and upload the image file from the PC to the server, having entered an authorized username and a given password. To add spot information, the user clicks the button marked "Detect the spot position" after placing the mouse cursor on the spot in the gel image and clicking the left button of the mouse. Thereafter an empty list of spot information is displayed with a "clickable map" function. The positional data for the spot are entered in the RDB table in the LIMS automatically by this operation. To update the spot information, the users press the left button of the mouse while the cursor is in the rectangle indicating the spot on the gel. Thereafter the spot information is displayed on the monitor of the client PC.

**Figure 4:**
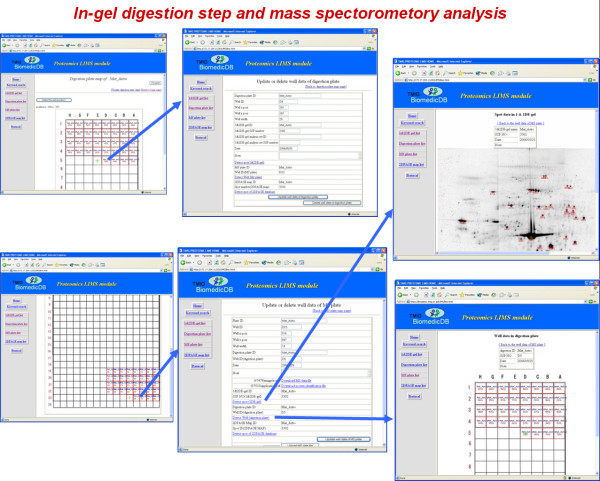
**The graphic user interface for in-gel digestion steps and mass spectrometry analyses**. LIMS users are allowed to operate the experimental data through the web interface. They are able to use MS plate list window, digestion plate list window, user login window, MS plate image window, digestion plate image window, well information window and etc for in-gel digestion steps and mass spectrometry analysis.

Figure 4 shows the GUI flow entering the well information for the digestion plate and the MS plate. First the user must select the type of plate and enter the plate information. The well information is entered on illustrations of the digestion plate and the MS plate. To add the well information, the user clicks the button marked "Detect the well position" after placing the mouse cursor on the well illustration of the digestion plate or the MS plate and clicking the left button of the mouse. The well ID is entered in the RDB table in the LIMS automatically by this operation. To update the well information, the user places the mouse cursor on the well with the red rectangle in the illustration, and clicks the left button of the mouse. Thereafter the well information is displayed.

While entering the corresponding SSP number and the corresponding well IDs in the digestion plate and the MS plate, the user can move and browse the various steps of proteomics workflow by clicking the linked button. If it is necessary to change the SSP number after the entering the data for some steps, the user enters the reference SSP number (SSP number of analysis set). The user is allowed to use the reference SSP number in the map for the public database.

Only the administrator of the LIMS can add the user and his attribution using the web interface. Only individual users can change their respective passwords. The LIMS does not have a special interface for entering the attribution of the administrator and the types of plates. Only the administrator of the PostgreSQL can enter them with the sql command on the server console.

### 3. Database tables

**Figure 5:**
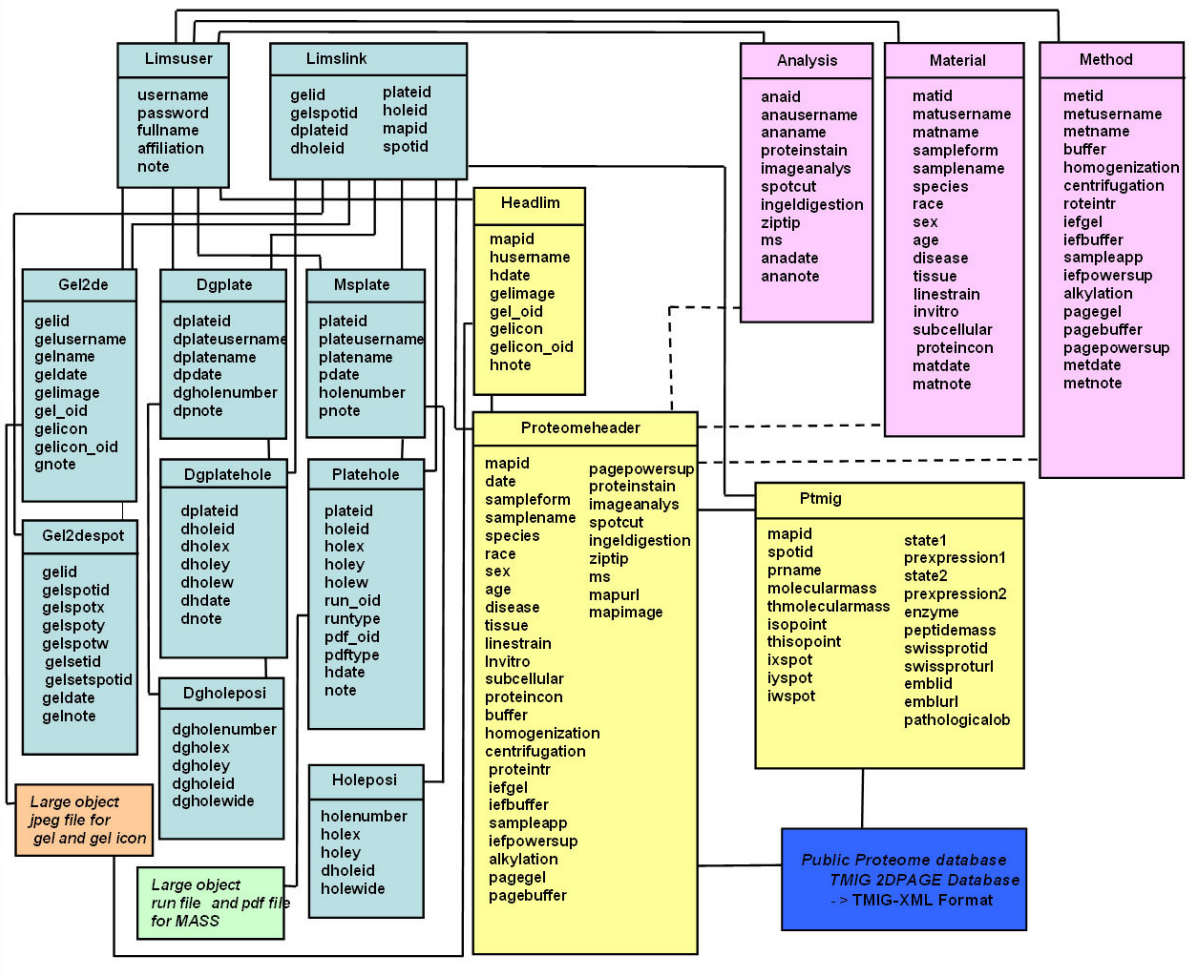
**Data tables of the LIMS**. We design the data tables for PostgreSQL in our LIMS for achieving compactness, flexibility, and connectivity to public databases.

**Figure 6:**
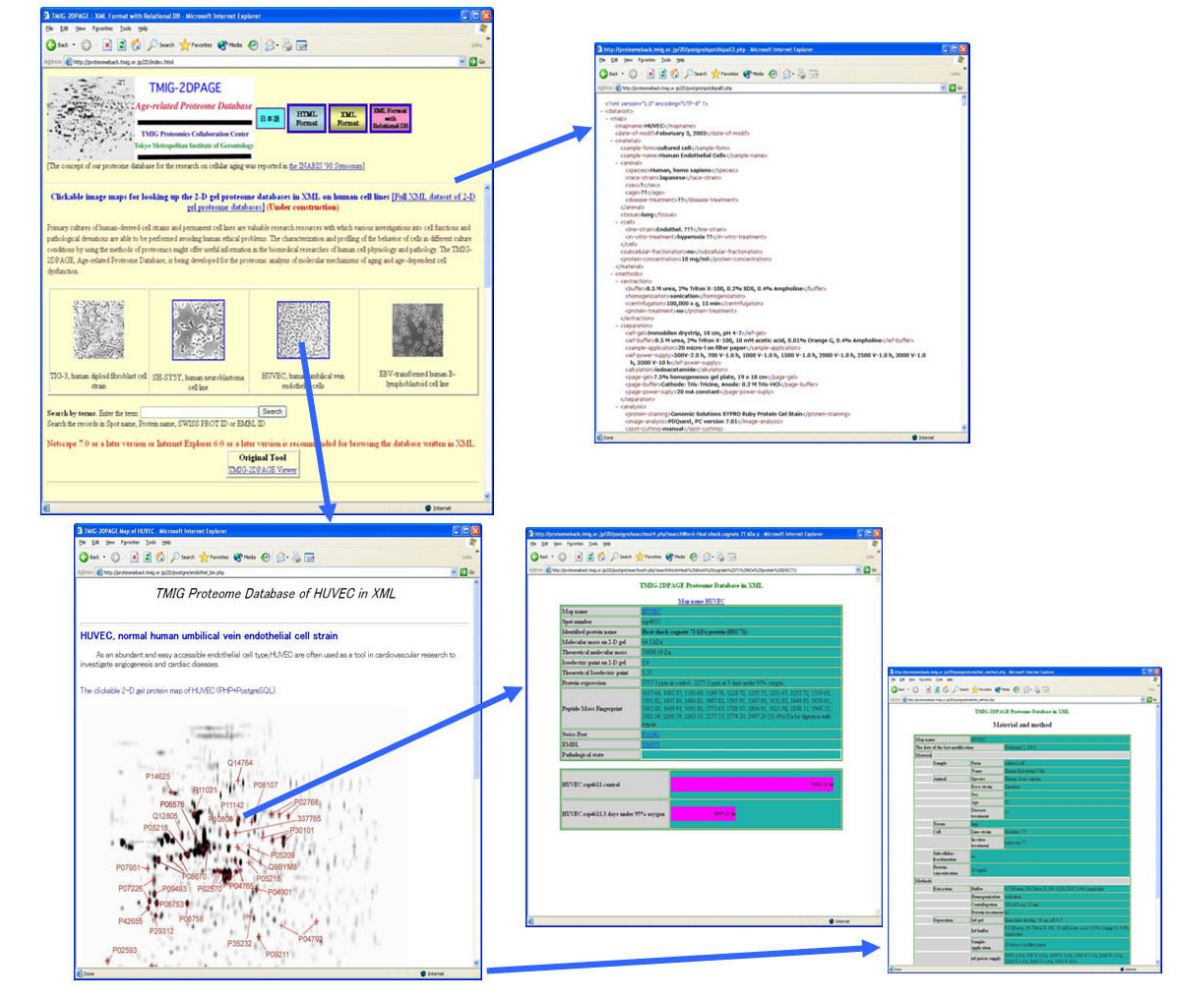
**TMIG-2DPAGE database : public proteome database in the Internet**. We had been publishing 2DPAGE database in the Internet since1996. The concept of our proteome database is for promoting aging researches. So, quantitative data of protein spots that are related to aging mechanisms are also included in the database.

Figure 5 shows the RDB tables for the LIMS. The table for "Limslink" includes linkages between the tables for "Gel2despot", "Dgplatehole" and "Platehole" to overwrite or delete. The tables for "Gel2de", "Headlim" and "Platehole" have an object identifier (oid) of large objects for raw data files. The table for "Platehole" has raw data-type files. The kinds of raw data file for mass spectrometry analysis are not restricted in the LIMS. The tables for "Holeposi" and "Dgholeposi" include the linkages between the well ID in the plates and the location of their illustration. They control the graphical interface with the illustration of the plates. The tables for "Proteomeheader" and "Ptmig" of the TMIG-2DPAGE database have the original contents and copies of the other LIMS tables. The TMIG-2DPAGE database has another copy of the tables and the JPEG image files exported from the LIMS. The contents of the TMIG-2DPAGE database must be certified in the LIMS (Figure 6).

## Results

We have developed an open source LIMS, which is compatible with a variety of data, formats and data sizes for current proteomic research. The data in the LIMS including raw files work as a backup of personal experimental data for permanent storage. Users are able to store their data with a certain degree of security. We have also encoded scripts for dumping and transferring all of the data automatically to a data backup server via Ethernet by FTP.

We have achieved a common "look and feel" in the LIMS. The "look and feel" that is designed with buttons and illustrations in the user interface supports user-friendly operation. Development of a LIMS featuring the above concepts is quite challenging because the 2-D gel electrophoresis-based workflow varies among laboratories.

Our intention was to develop a compact "personal" LIMS that is appropriate for small laboratories. Our LIMS can be customized easily by any laboratory. The LIMS we have developed may be a practical tool for proteome researchers at any institutions where 2-D gel-electrophoresis-based proteomics research is being conducted.

## Discussion

We have developed an open source LIMS optimized for proteomics after completion of the development of a commercial base LIMS, WorksBase, by Bio-Rad Laboratories Inc. We have experienced the experimental operation of WorksBase, which is an integrated bioinformatics platform for 2-D gel electrophoresis-based proteomics. In view of the severe competition that exists in the field of life science research, both security and perfection of the experimental information in WorksBase were considered important. However, data management using WorksBase was still troublesome for some reason or other. We discussed the specifications and problems inherent to WorksBase and designed our own LIMS based on our experience with its operation. We considered that simplicity and usefulness were more important than perfection for a LIMS in our laboratory. The major features of our LIMS are compactness, flexibility and compatibility with the public database.

Many proteomics researchers have been awaiting the development of a LIMS for 2-D gel electrophoresis-based workflow. Up to now, however, most commercial LIMS have not supported 2-D gel electrophoresis-based workflow because the concept is more complex than other workflows employed in proteomics LIMS. Therefore we designed our LIMS to specifically support 2-D gel electrophoresis-based proteomics workflow. Consequently, the content of our LIMS is not satisfactory for allowing all proteomics researchers to manage all proteomic information properly. We think that the content of the standardized format established by HUPO-PSI is appropriate for proteomics in general, but the linkages within it are too complex for our LIMS. Thus we were unable to organize these linkages, which had been established in XML format, in our LIMS. We intend to develop a LIMS without a XML native database to allow more rapid use. We would like to further improve the LIMS in order to support the contents of HUPO-PSI with the function of conversion.

**Figure 7:**
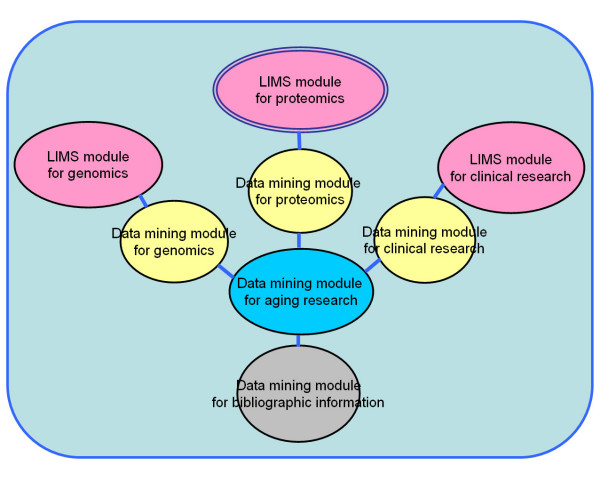
**The LIMS for proteomics as a module of our biomedical informatics system (Biomedic DB)**. In this system, LIMS modules for genomics, proteomic and clinical research are constructed separately. The data mining modules will be connected to LIMS modules through the individual data mining modules for genomics, proteomics and clinical research.

**Figure 8:**
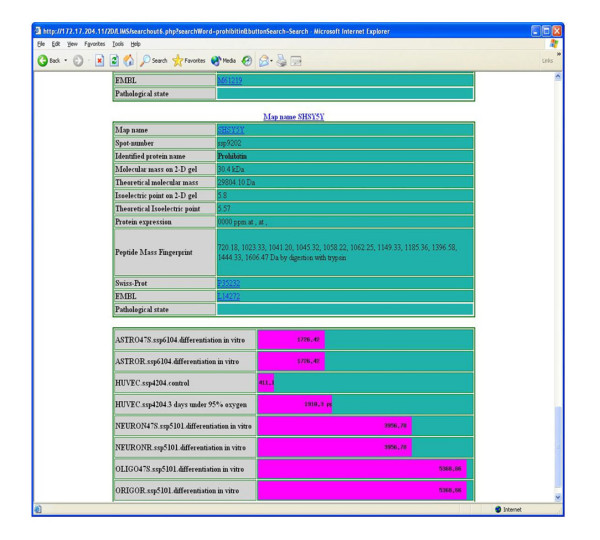
**The bar graph showing the relative abundance in TMIG-2DPAGE database**. TMIG-2DPAGE database and our LIMS are designed to display the bar graph indicating the relative abundance of the corresponding spots of the 2DPAGE gel automatically.

Currently, many proteomics researchers are eager to use effective data-mining tools for analysis of proteomic data. However, we think that our proteomics LIMS should be developed as a module separate from the data-mining tools in our "Bio-Medic DB" bioinformatics platform that will be constructed in the future (Figure 7). We are planning to develop a multi-module system, "Bio-Medic DB", that will be composed of a LIMS module and a data-mining module for proteome, genome and clinical research at our institution. On the other hand, we can assay relative abundance in the form of a bar graph by searching the TMIG-2DPAGE database with key words, which is calculated by copies of the LIMS contents (Figure 8). Both private and public tools for proteomics data-mining need to be discussed.

We are also planning to develop a new interface of the LIMS for XML format files exported from PDQuest (Bio-Rad Laboratories Inc.). PDQuest compares 2D gel images to determine differential protein expression. We intend to develop special client software running a data-upload function in Windows XP because the XML format files exported from PDQuest are too complex for web applications.

In 2005, Garden, Alm and Hakkinen developed PROTEIOS: an open source proteomics initiative [16]. PROTEIOS is implemented in Java and SQL. It is a client-server open source application for proteomics. However, our LIMS was originally developed and distributed under the GNU General Public License, which means that its source code is freely distributable and available to the general public. Everybody can download the source code on the basis of the requirements listed below.

## Conclusion

We have developed a basic model for an open source LIMS that is effective for 2-D gel electrophoresis-based proteomics workflow. We expect that the open source LIMS will be a powerful tool in advance proteome researches in many small laboratories. We hope many proteomics researchers to download and use our open source LIMS, and wish to receive feedback about their experience in operating it in order to draw up guidelines for a proteomics LIMS. Please see the additional file that includes all PHP scripts, sql and html files of our LIMS [see Additional file 1].

## Availability and requirements

. Project home page:  by following the web link.

. Operating system: Red Hat Linux 9

. Programming language: PHP, PostgreSQL

. Requirements: Apache revision 1.3.34 or later, PostgreSQL revision 7.4.3 or later, PHP revision 4.3.7 or later

. License: Lesser General Public License

The source file of the new LIMS for proteomics can be accessed using a web browser at  by following the web link.

## Abbreviations

LIMS, Laboratory information management system: XML, Extensible markup language: PHP, Hypertext Preprocessor: 2-D PAGE, Two-dimensional polyacrylamide gel electrophoresis: MS, Mass spectrometry: TMIG, Tokyo Metropolitan Institute of Gerontology: RDB, Relational database: SSP number, Standard spot number

## Supplementary Material

Additional File 1 **Our program of LIMS**. The file is a compressed file that includes all PHP scripts, sql and html files of our LIMS. Please install Apache revision 1.3.34 or later, PostgreSQL revision 7.4.3 or later, PHP revision 4.3.7 or later and GD library revision 2.0.27 or later in advance of setting up the LIMS. The LIMS is licensed under GNU Lesser General Public License. Please set up as follows. tar zxvf LIPAGE_0.88.tar.gz. mv LIMS/usr/local/apache/htdocs. Please read/usr/local/apache/htdocs/LIMS/README. Click here for file

Additional File 2 **The documentation of simple usage for our LIMS**. The file is a documentation of simple usage for our LIMS. Click here for file
