# Supplementary material for: Development of an open source laboratory information management system for 2-D gel electrophoresis-based proteomics workflow
Source: BMC Bioinformatics. 2006 Oct 4;7:430. doi: 10.1186/1471-2105-7-430 (PMC1599757; doi:10.1186/1471-2105-7-430)
Supplement: Additional File 1 — Our program of LIMS. The file is a compressed file that includes all PHP scripts, sql and html files of our LIMS. Please install Apache revision 1.3.34 or later, PostgreSQL revision 7.4.3 or later, PHP revision 4.3.7 or later and GD library revision 2.0.27 or later in advance of setting up the LIMS. The LIMS is licensed under GNU Lesser General Public License. Please set up as follows. tar zxvf LIPAGE_0.88.tar.gz. mv LIMS/usr/local/apache/htdocs. Please read/usr/local/apache/htdocs/LIMS/README. [file 1471-2105-7-430-S1.gz › LIMS/dspotposi.php]

Add well data of digestion plate 


Add well data of digestion plate 
php print("<TABLE|  | \n"); print("\n"); print("[Back to digestion plate map page] |
\n"); ?>

---

php print("<TR Digestion plate ID |  |\n"); ?>
php
$connectDbName = "host=localhost port=5432 dbname=proteomedb";
$dbHandle = pg\_connect($connectDbName);
if ($dbHandle == FALSE) {
print("can not connect database<BR\n");
exit;
}
$sql10 = "select dgholenumber ,dgholex ,dgholey ,dgholeid, dgholewide from dgholeposi "
."where dgholenumber = '$dgholenumber'";
$result10 = pg\_exec($dbHandle, $sql10);
if ($result10 == 0) {
print("SQL10:\"$sql10\"can not exec");
pg\_close($dbHandle);
exit;
}
$resultNumRows10 = pg\_numrows($result10);
if($resultNumRows10 > 0){
$rowCount10 = 0;
while ($rowCount10 < $resultNumRows10) {
$data10 = pg\_fetch\_object($result10, $rowCount10);
$updgholex2 = $data10->dgholex;
$updgholey2 = $data10->dgholey;
if ($point\_x <= $updgholex2+$updgholew2 and $point\_x >= $updgholex2-$updgholew2 and $point\_y <= $updgholey2+$updgholew2 and $point\_y >= $updgholey2-$updgholew2)
{ $updgholeid = $data10->dgholeid;}
$rowCount10++;
}
} pg\_free\_result($result10);
?>
php print("<TR Well ID(necessary) |  |\n"); ?>
php print("<TR Well x pos |  |\n"); ?>
php print("<TR Well Y pos |  |\n"); ?>
php print("<TR Well width |  |\n"); ?>| 1&2DE-gel name |  |
| 1&2DE-gel SSP number |  |
| 1&2DE-gel analysis set name |  |
| 1&2DE-gel analysis set SSP number |  |
php $datestamp = date("Y/n/j");
print("<TR Date |  |\n"); ?>| Note | php print("<textarea NAME=\"dnote\" rows=\"2\" cols=\"50\" wrap=\"soft\" |
\n");
print("\n");
print("\n"); ?>|  | |
