# Supplementary material for: Development of an open source laboratory information management system for 2-D gel electrophoresis-based proteomics workflow
Source: BMC Bioinformatics. 2006 Oct 4;7:430. doi: 10.1186/1471-2105-7-430 (PMC1599757; doi:10.1186/1471-2105-7-430)
Supplement: Additional File 1 — Our program of LIMS. The file is a compressed file that includes all PHP scripts, sql and html files of our LIMS. Please install Apache revision 1.3.34 or later, PostgreSQL revision 7.4.3 or later, PHP revision 4.3.7 or later and GD library revision 2.0.27 or later in advance of setting up the LIMS. The LIMS is licensed under GNU Lesser General Public License. Please set up as follows. tar zxvf LIPAGE_0.88.tar.gz. mv LIMS/usr/local/apache/htdocs. Please read/usr/local/apache/htdocs/LIMS/README. [file 1471-2105-7-430-S1.gz › LIMS/limsadmin.html]

Adminstrate 

*Add or delete user* 


|  |  |
| --- | --- |
| *Administrator name* |  |
| *Password* |  |
|  |  |

 
