# Supplementary material for: Development of an open source laboratory information management system for 2-D gel electrophoresis-based proteomics workflow
Source: BMC Bioinformatics. 2006 Oct 4;7:430. doi: 10.1186/1471-2105-7-430 (PMC1599757; doi:10.1186/1471-2105-7-430)
Supplement: Additional File 1 — Our program of LIMS. The file is a compressed file that includes all PHP scripts, sql and html files of our LIMS. Please install Apache revision 1.3.34 or later, PostgreSQL revision 7.4.3 or later, PHP revision 4.3.7 or later and GD library revision 2.0.27 or later in advance of setting up the LIMS. The LIMS is licensed under GNU Lesser General Public License. Please set up as follows. tar zxvf LIPAGE_0.88.tar.gz. mv LIMS/usr/local/apache/htdocs. Please read/usr/local/apache/htdocs/LIMS/README. [file 1471-2105-7-430-S1.gz › LIMS/limssearch.htm]

TMIG-2D LIMS MAIN


### Keyword search

---

|  |  |
| --- | --- |
| **Search 1 & 2DE-gels for spot data** | |
| Enter the term : |  |
|  | SSP number, note, linked well ID etc. |
|  | |
|  |  |  |  |  |  |
| --- | --- | --- | --- | --- | --- |
| **Search digestion plates for well data**| Enter the term : |  | |  | Well ID, note, linked SSP number etc. | | |
|  | |
|  |  |  |  |  |  |
| --- | --- | --- | --- | --- | --- |
| **Search MS plates for well data** | Enter the term : |  | |  | Well ID, note, linked SSP number etc. | | |
|  | |
|  |  |
| --- | --- |
| **Search 2DPAGE database for spot data** | |
| Enter the term : |  |
|  | Spot number, protein name, swissprot ID etc. |

| **Search 2DPAGE database for spot data (graph of protein expression)** | |
|  |  |
| --- | --- |
| Enter the term : |  |
|  | Spot number, protein name, swissprot ID etc. ||
