# Supplementary material for: Development of an open source laboratory information management system for 2-D gel electrophoresis-based proteomics workflow
Source: BMC Bioinformatics. 2006 Oct 4;7:430. doi: 10.1186/1471-2105-7-430 (PMC1599757; doi:10.1186/1471-2105-7-430)
Supplement: Additional File 1 — Our program of LIMS. The file is a compressed file that includes all PHP scripts, sql and html files of our LIMS. Please install Apache revision 1.3.34 or later, PostgreSQL revision 7.4.3 or later, PHP revision 4.3.7 or later and GD library revision 2.0.27 or later in advance of setting up the LIMS. The LIMS is licensed under GNU Lesser General Public License. Please set up as follows. tar zxvf LIPAGE_0.88.tar.gz. mv LIMS/usr/local/apache/htdocs. Please read/usr/local/apache/htdocs/LIMS/README. [file 1471-2105-7-430-S1.gz › LIMS/mapspotposi.php]

TMIG-2D XML DATABASE DATA ADD


Add Spot Data ( 2DPAGE map)
php print("<TABLE|  | \n"); print("\n"); print("[Back to 2DPAGE map page] |
\n"); ?>

---

php print("<TR Map name |  |\n"); ?>| Spot number (necessary) |  |
| Protein name (necessary) |  |
| Mol. mass |  |
| Theoretical mol. mass |  |
| pI |  |
| Theoretical pI |  |
php print("<TR Spot x pos |  |\n"); ?>
php print("<TR Spot Y pos |  |\n"); ?>
php print("<TR Spot width |  |\n"); ?>| Prot. expression 1 |  |
| at state 1 |  |
| Prot. expression 2 |  |
| at state 2 |  |
| Enzyme |  |
| Peptide mass |  |
| Swissprot id |  |
| Swissprot url |  |
| EMBL id |  |
| EMBL url |  |
| Pathological info. |  |
| MS plate ID |  |
| Well ID |  |
|  | |
php print("<input type=\"hidden\" name=\"username\" value=$username\n");
print("\n"); ?>|  | |
