# Supplementary material for: Development of an open source laboratory information management system for 2-D gel electrophoresis-based proteomics workflow
Source: BMC Bioinformatics. 2006 Oct 4;7:430. doi: 10.1186/1471-2105-7-430 (PMC1599757; doi:10.1186/1471-2105-7-430)
Supplement: Additional File 1 — Our program of LIMS. The file is a compressed file that includes all PHP scripts, sql and html files of our LIMS. Please install Apache revision 1.3.34 or later, PostgreSQL revision 7.4.3 or later, PHP revision 4.3.7 or later and GD library revision 2.0.27 or later in advance of setting up the LIMS. The LIMS is licensed under GNU Lesser General Public License. Please set up as follows. tar zxvf LIPAGE_0.88.tar.gz. mv LIMS/usr/local/apache/htdocs. Please read/usr/local/apache/htdocs/LIMS/README. [file 1471-2105-7-430-S1.gz › LIMS/spotposi.php]

TMIG-2D LIMS Plate Hole Data Add


Add well data of MS plate
php print("<TABLE|  | \n"); print("\n"); print("[Back to MS plate map page] |
\n"); ?>

php print("<TR MS plate ID |  |\n"); ?>
php
$connectDbName = "host=localhost port=5432 dbname=proteomedb";
$dbHandle = pg\_connect($connectDbName);
if ($dbHandle == FALSE) {
print("can not connect database<BR\n");
exit;
}
$sql10 = "select holenumber ,holex ,holey ,dholeid, holewide from holeposi "
."where holenumber = '$holenumber'";
$result10 = pg\_exec($dbHandle, $sql10);
if ($result10 == 0) {
print("SQL10:\"$sql10\"can not exec");
pg\_close($dbHandle);
exit;
}
$resultNumRows10 = pg\_numrows($result10);
if($resultNumRows10 > 0){
$rowCount10 = 0;
while ($rowCount10 < $resultNumRows10) {
$data = pg\_fetch\_object($result10, $rowCount10);
$upholex2 = $data->holex;
$upholey2 = $data->holey;
if ($point\_x <= $upholex2+$upholew2 and $point\_x >= $upholex2-$upholew2 and $point\_y <= $upholey2+$upholew2 and $point\_y >= $upholey2-$upholew2)
{ $updholeid = $data->dholeid; }
$rowCount10++;
}
} pg\_free\_result($result10);
?>
php print("<TR Well ID(necessary) |  |\n"); ?>
php print("<TR Well x pos |  |\n"); ?>
php print("<TR Well Y pos |  |\n"); ?>
php print("<TR Well width |  || Digestion plate ID |  |
| Well ID of digestion plate |  |
\n"); ?>
php $datestamp = date("Y/n/j");
print("<TR Date |  |\n");
print("| Note |  |
\n"); ?>

php
print("<input type=\"hidden\" name=\"username\" value=$username\n");
print("\n"); ?>

|  |  |
| --- | --- |
|  |  |
