# Supplementary material for: Development of an open source laboratory information management system for 2-D gel electrophoresis-based proteomics workflow
Source: BMC Bioinformatics. 2006 Oct 4;7:430. doi: 10.1186/1471-2105-7-430 (PMC1599757; doi:10.1186/1471-2105-7-430)
Supplement: Additional File 2 — The documentation of simple usage for our LIMS. The file is a documentation of simple usage for our LIMS. [file 1471-2105-7-430-S2.pdf]

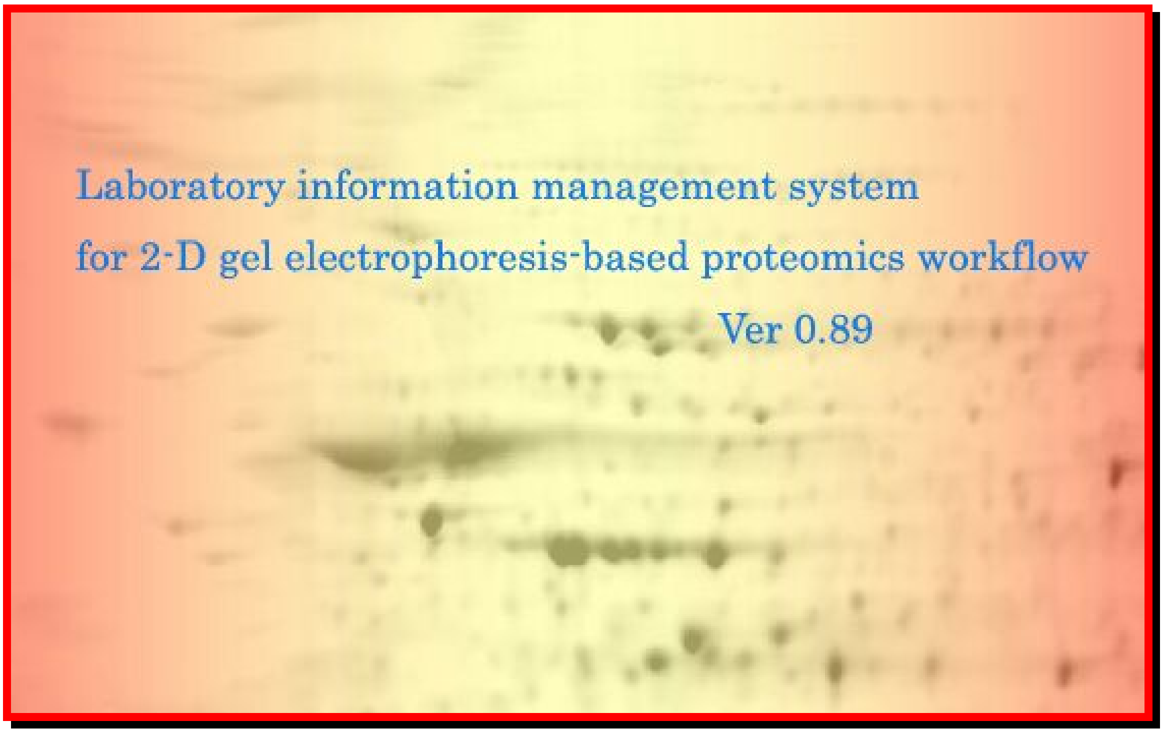

Simple Usage

Served by Proteomics Collaboration Research Group,
Tokyo Metropolitan Institute of Gerontology
35-2 Sakae-cho, Itabashi-ku, Tokyo, 173-0015, Japan
E-mail : proteomi@tmig.or.jp
tel : 03-3964-3241

Index

Chapter 1. Introduction --2

Chapter 2. Add 1&2DE-gel data, digestion plate data, MS plate and 2DEPAGE map data --2

Chapter 3. Add and update protocol data --9

Chapter 4. Component parts --22
- 4-1. Home
- 4-2. Keyword search
- 4-3. Editing workflow data
- 4-4. Protocol data
- 4-5. Change user password
- 4-6. Administrator only

Chapter 5. Installation --37
- 5-1 Setup
- 5-2 Apache-PHP-PostgreSQL installation instruction using source files
- 5-3 Apache-PHP-PostgreSQL installations instruction using rpm packages on Fedora Core 4 and 5

Chapter 1 Introduction

We developed an open source laboratory information management system for 2-D gel electrophoresis-based proteomics workflow. The software development project is served by Proteomics Collaboration Research Group members, Tokyo Metropolitan Institute of Gerontology. This new LIMS is licensed under GNU Lesser General Public License. The LIMS is carried out as PostgreSQL-PHP-apache system on Linux OS. We explain how to use the LIMS.
In order to begin with this program, open the Poteomics LIMS module window of your PC. This Poteomics LIMS system can be used only by a registered user inputting "User name" and "Password". This is the system which creates a synthetic analysis by inputting user's research data and related information to each unit.

Chapter 2 Add 1&2DE-gel data, Digestion plate data, MS plate and 2DEPAGE map data

Open the window of "Home" including "Add or update 1&2DE-gel data", "Add or update digestion plate data", "Add or update MS plate data" and "Add or update 2DPAGE map data".

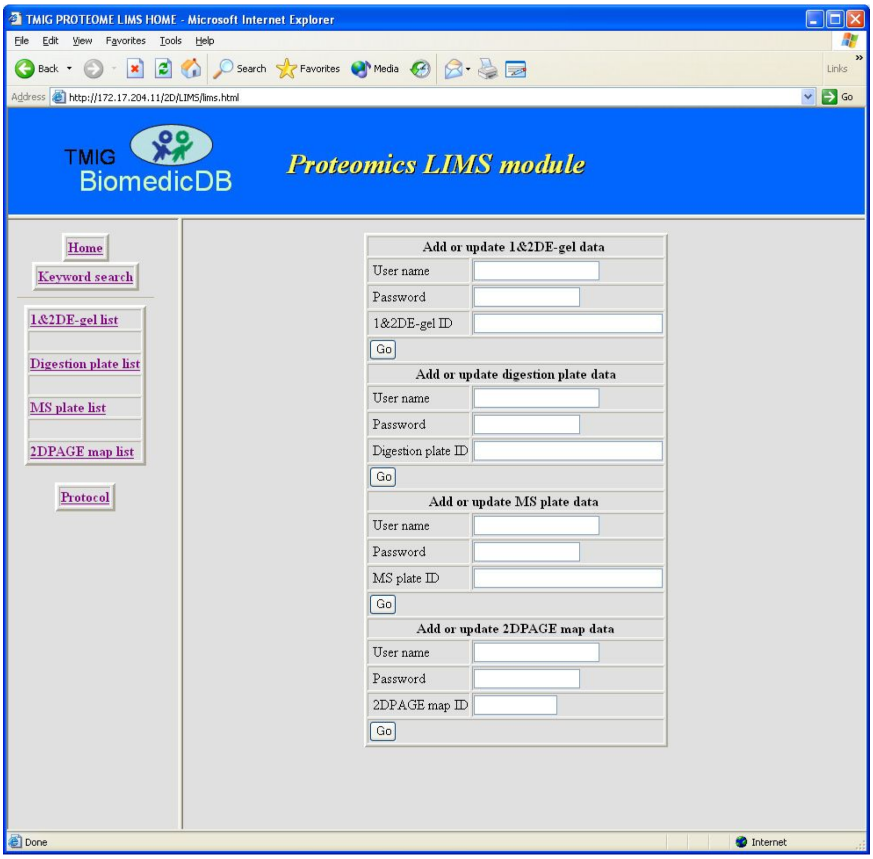

Fig.1

At first, enter the User name (ex. morisawa), Password (****) and 1&2-gel ID (ex. mori_test) in each box, and click on "Go" button (Fig.1). Then the window of "Add 1&2DE-gel data of mori_test" appears. In this window, enter "1&2DE-gel name (ex. mori_test gel)", and click on "Add new gel data" button (Fig.2). Then the window of "Saved data is OK!" appears, and click on "Upload gel image file or gel image icon file"

link button (Fig.3). Then the window of "Update or delete 2DE-gel data of mori_test" appears. In this window, input experimental data to each item, and upload 2DE-gel image and 2DE-gel icon. 2DE-gel icon (width:120 pixel) must be prepared by users. Uploading a TIFF image instead of a JPEG image causes an error. Check the uploaded gel image and gel icon (Fig.4).

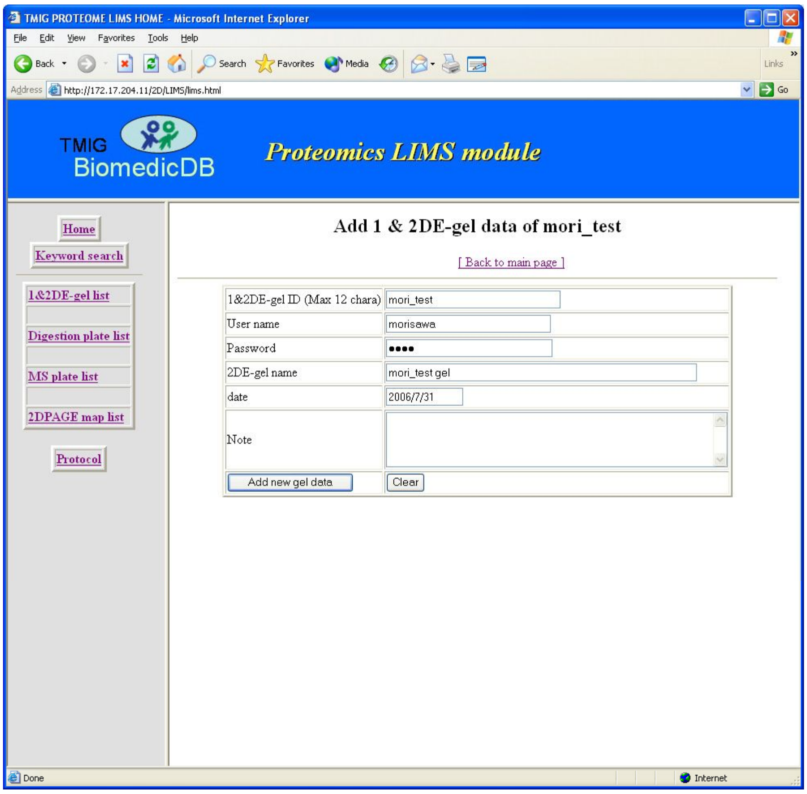

Fig.2

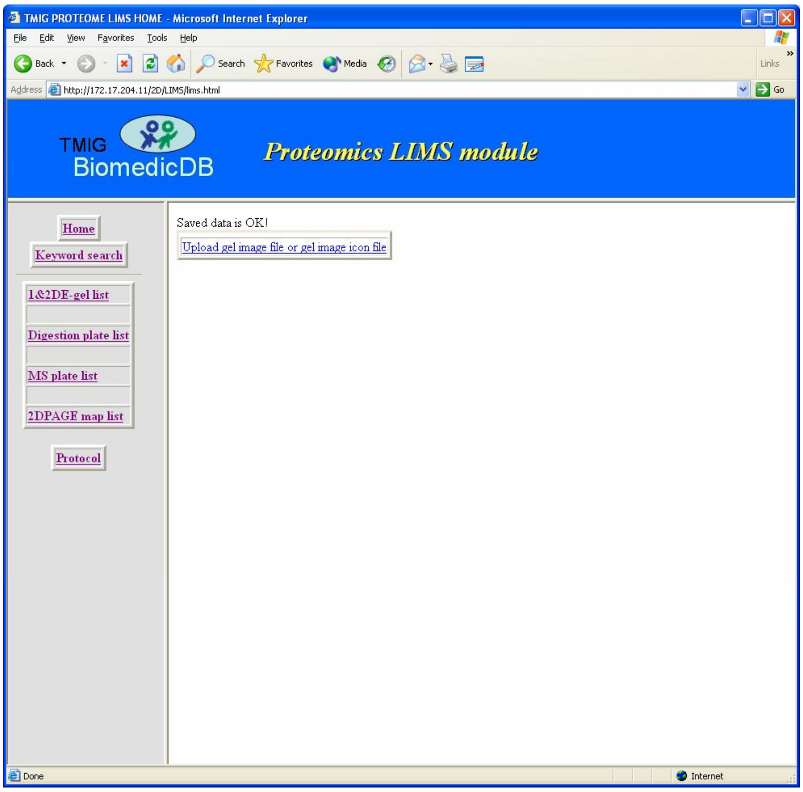

Fig.3

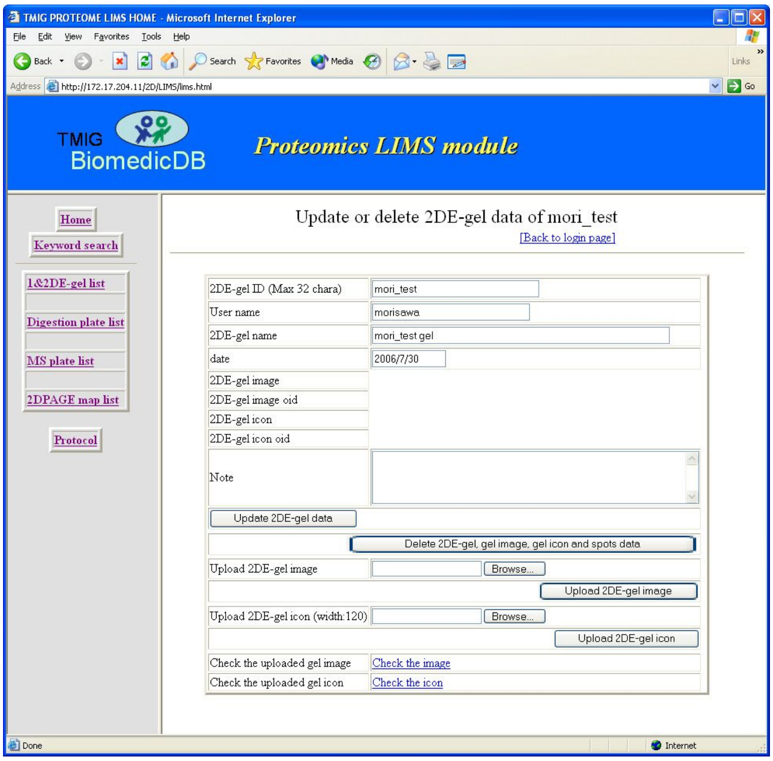

Fig.4

Secondly in the "Home" window, enter the User name (ex. morisawa), Password (****) and Digestion plate ID (ex. mori_test) in each box, and click on "Go" button (Fig.1). Then the window of "Add digestion plate data of mori_test" appears. In this window, enter the each item, and click on "Add digestion plate data" button (Fig.5). Then the window of "Digestion plate map of mori_test" appears (Fig.6).

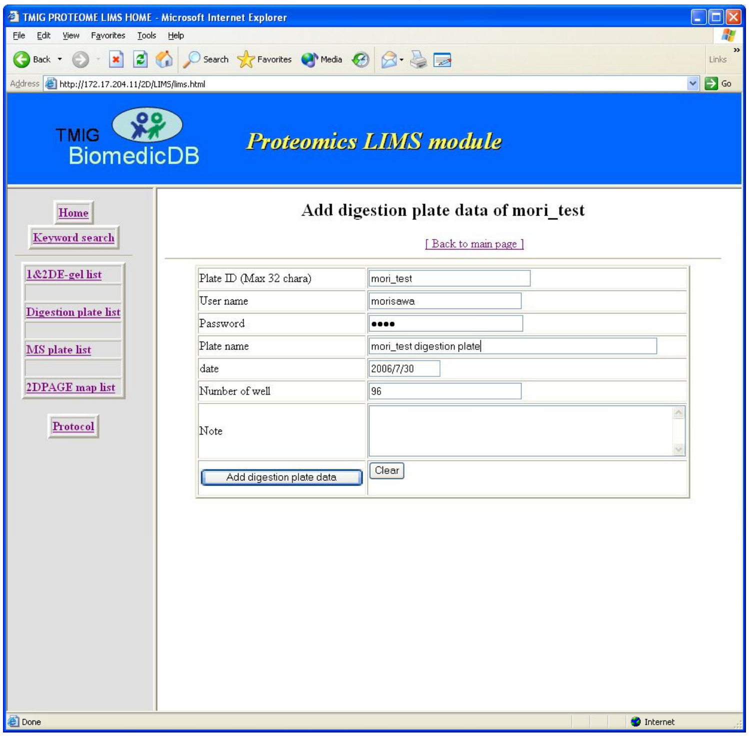

Fig.5

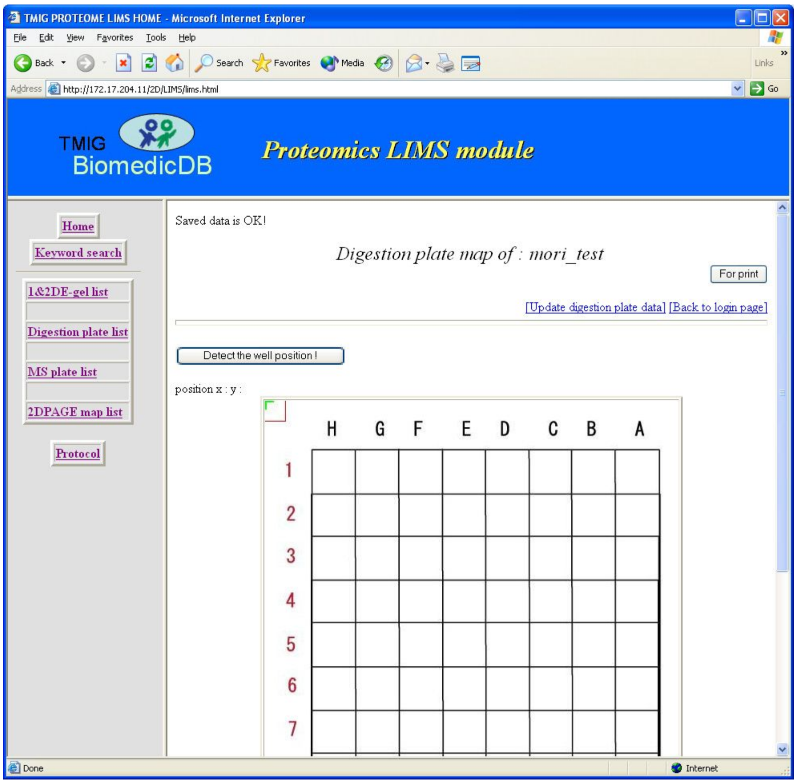

Fig.6

Thirdly in the "Home" window, enter the User name (ex. morisawa), Password (****) and MS plate ID (ex. mori_test) in each box, and click on "Go" button (Fig.1). Then the window of "Add MS plate data of mori_test" appears. In this window, enter the each item, and click on "Add MS plate data" button (Fig.7). Then the window of "MS plate map of mori_test" appears (Fig.8).

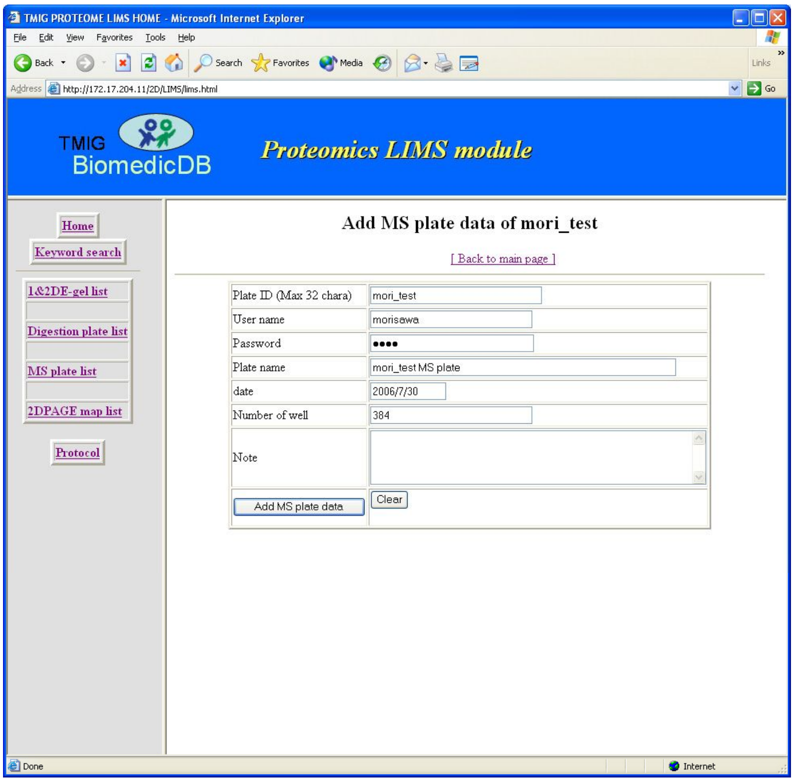

Fig.7

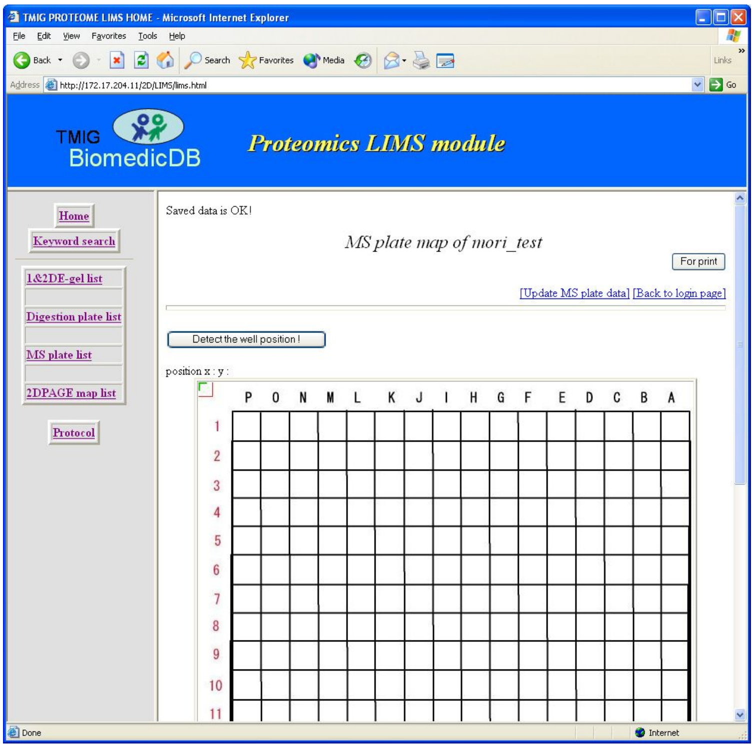

Fig.8

Finally in the "Home" window, enter the User name (ex. morisawa), Password (****) and 2DEPAGE map ID (ex. mori_test) in each box, and click on "Go" button (Fig.1). Then the window of "Add Header and Map data" appears. In this window, input experimental data to each item, and click on "Add header and map data of 2DEPAGE" button (Fig.9). Then the window of "The addition of header data is OK! The addition of map data is OK!" appears. In this window, click on "Update the header of 2DPAGE map" link button(Fig.10). Then the window of "Update header data of 2DPAGE map: mori_test" appears. In this window, enter the each item, and upload 2DE-gel image and 2DE- gel icon. Moreover, copy 2DE-Gel data by gel ID and 2DE-Gel data by analysis set ID. Check the uploaded gel image and gel icon (Fig.11).

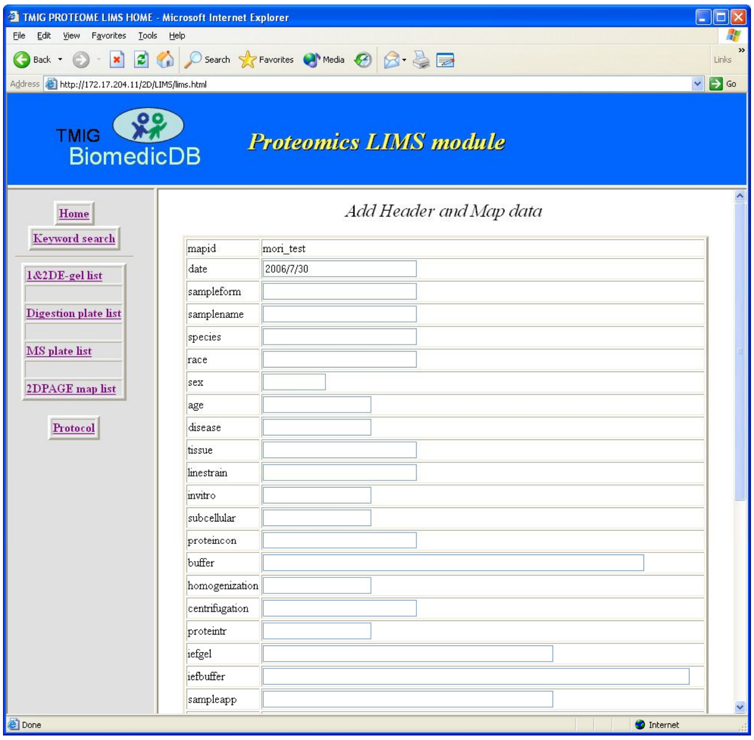
TMIG PROTEOME LIMS HOME - Microsoft Internet Explorer
http://172.17.204.11/2D/LIMS/lims.html
TMIG
BiomedicDB
Proteomics LIMS module
Home
Keyword search
1&2DE-gel list
Digestion plate list
MS plate list
2DPAGE map list
Protocol
Add Header and Map data
mapid
mori_test
date
2006/7/30
sampleform
samplename
species
race
sex
age
disease
tissue
linestrain
invitro
subcellular
proteincon
buffer
homogenization
centrifugation
proteintr
iefgel
iefbuffer
sampleapp

Fig.9

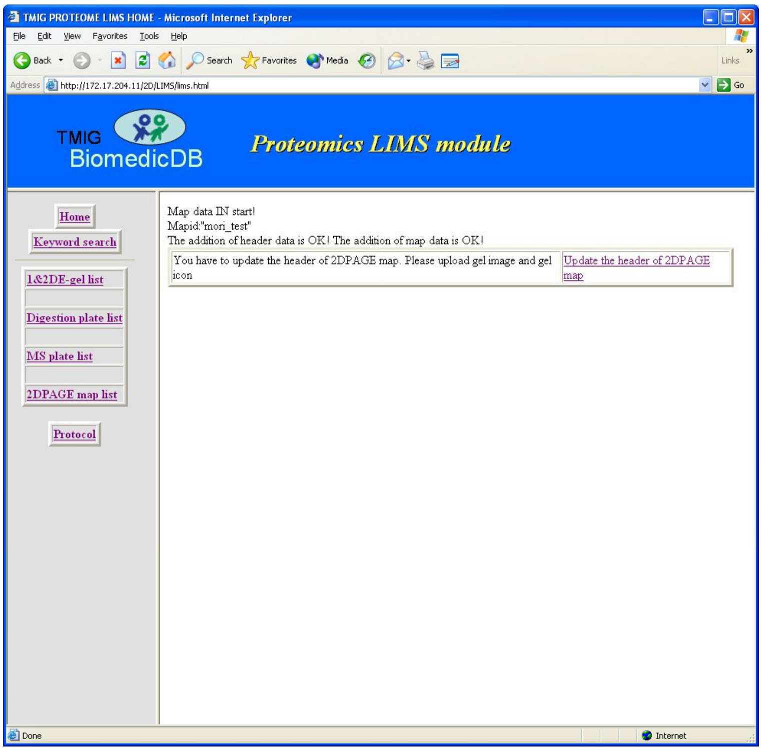
TMIG PROTEOME LIMS HOME - Microsoft Internet Explorer
http://172.17.204.11/2D/LIMS/lims.html
TMIG
BiomedicDB
Proteomics LIMS module
Home
Keyword search
1&2DE-gel list
Digestion plate list
MS plate list
2DPAGE map list
Protocol
Map data IN start!
Mapid:"mori_test"
The addition of header data is OK! The addition of map data is OK!
You have to update the header of 2DPAGE map. Please upload gel image and gel icon
Update the header of 2DPAGE map

Fig.10

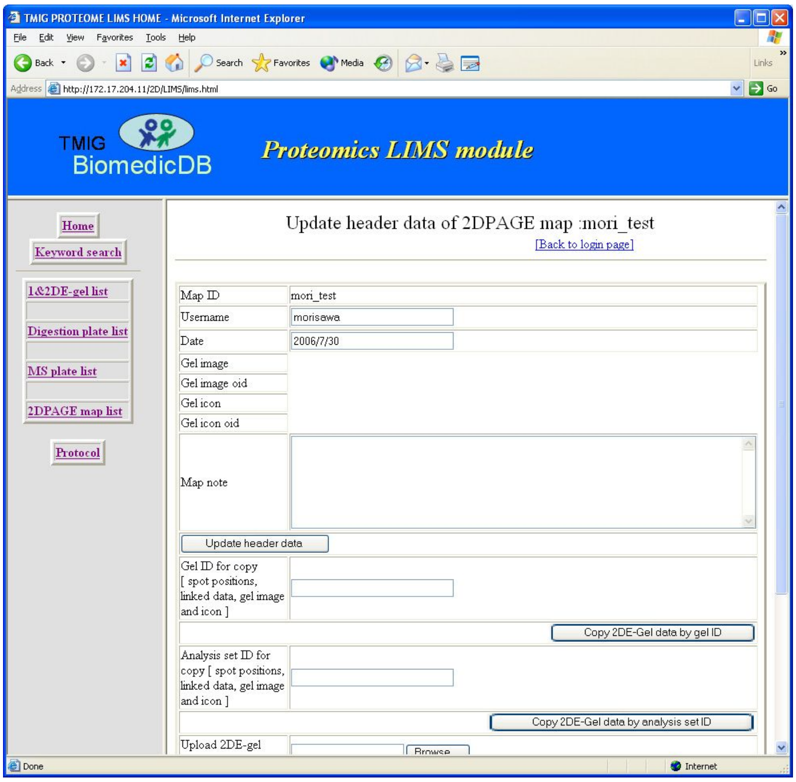
TMIG PROTEOME LIMS HOME - Microsoft Internet Explorer
http://172.17.204.11/2D/LIMS/lims.html
TMIG
BiomedicDB
Proteomics LIMS module
Home
Keyword search
1&2DE-gel list
Digestion plate list
MS plate list
2DPAGE map list
Protocol
Update header data of 2DPAGE map :mori_test
[Back to login page]
Map ID
mori_test
Username
morisawa
Date
2006/7/30
Gel image
Gel image oid
Gel icon
Gel icon oid
Map note
Update header data
Gel ID for copy [spot positions, linked data, gel image and icon]
Copy 2DE-Gel data by gel ID
Analysis set ID for copy [spot positions, linked data, gel image and icon]
Copy 2DE-Gel data by analysis set ID
Upload 2DE-gel
Browse...
Done
Internet

Fig.11

Chapter 3　Add and update protocol data

Open the window of “Protocol home” including ”Add or update material data”, ”Add or update gel method data” and ”Add or update analysis method data”.

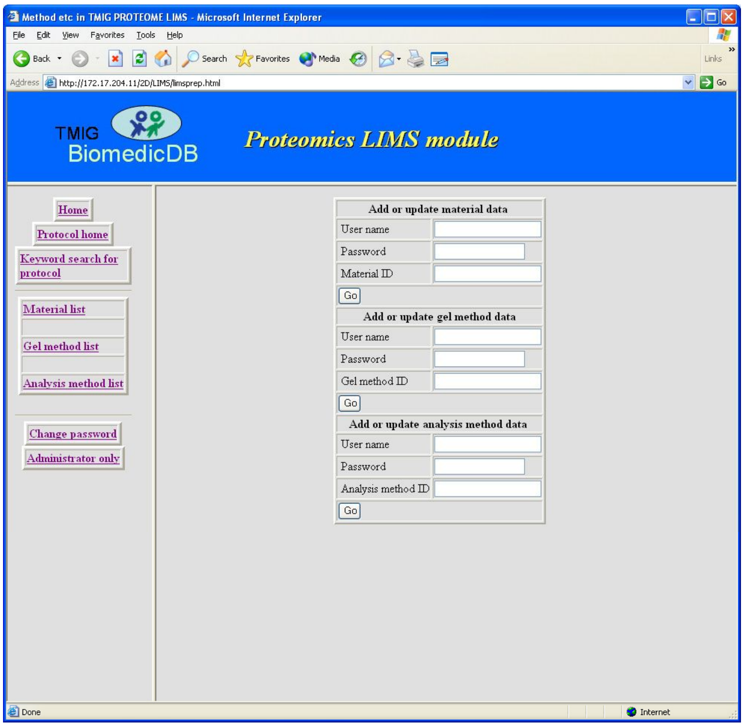

Fig.12

At first, enter the User name (ex. morisawa), Password (****) and Material ID (ex. mori_test) in each box, and click on “Go” button (Fig.12). Then the window of “Add material data of mori_test” appears. In this window, enter the each item, and click on “Add material data” button (Fig.13). Then the window of “The insert of data is OK!“ appears. In this window, click on “Material list” link button. Then the window of “Material list” appears.

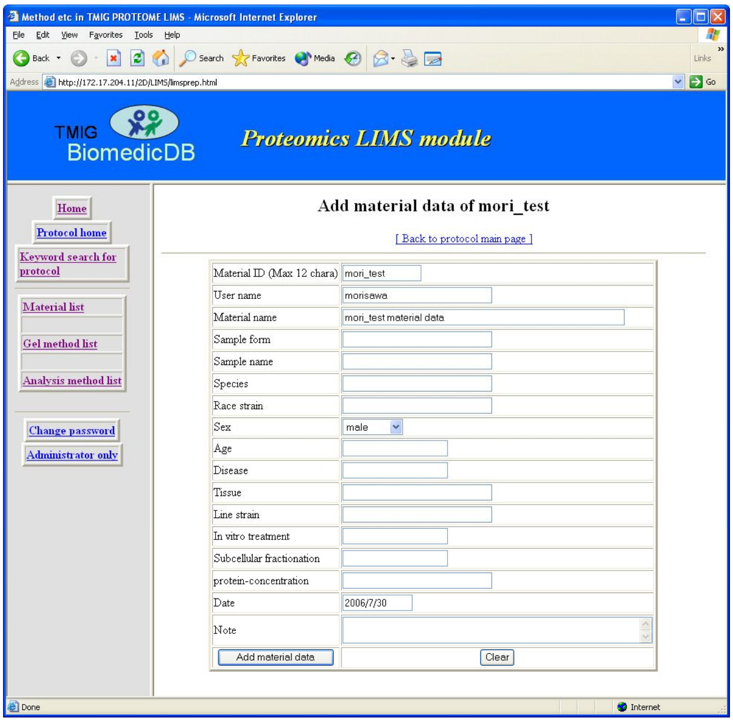

Fig.13

In this window, click on “Material ID (ex. Mat_Astro)” link button (Fig.14). Then the window of “Login for material” appears. Enter the Password (****), and click on “Update or delete material data “ button (Fig.15). Then the window of “Update or delete material data” appears. In this window, input experimental material data in each box, and update or delete material data (Fig.16). In addition, click on “Insert in 2DEPAGE map data” button, and then “2DPAGE map list” appears. In this window, click on the user’s gel icon (ex. Mat_Astro) (Fig.17). Then the window of “Login for 2DPAGE map (the insert of material data)” appears. Enter the Password (****), and click on “Update 2DPAGE map data (the insert of material data)” button (Fig.18). Then the window of “Update Map Data of Mat_Astro” appears. Input the data in each box, and click on “Update 2DPAGE map data” (Fig.19).

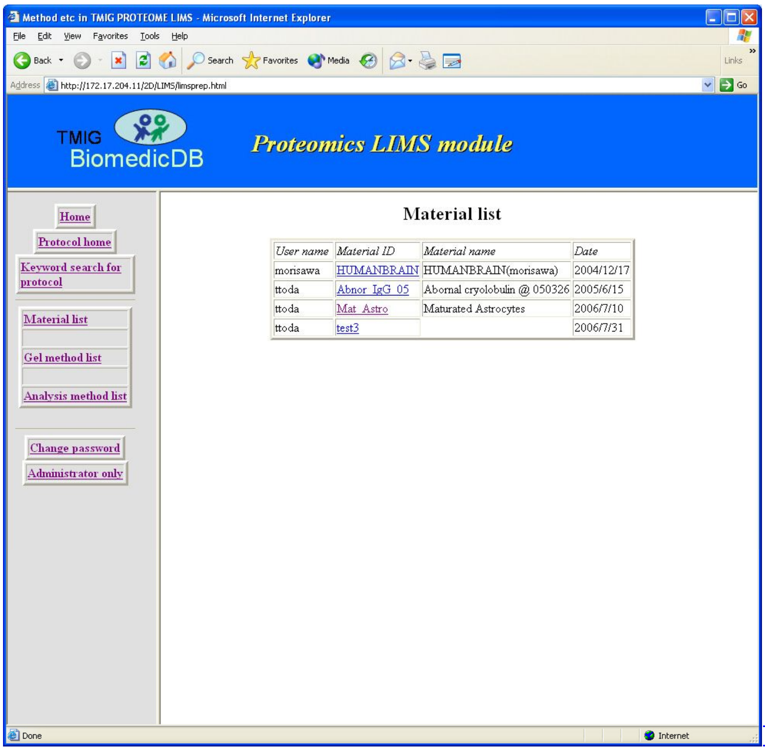

Fig.14

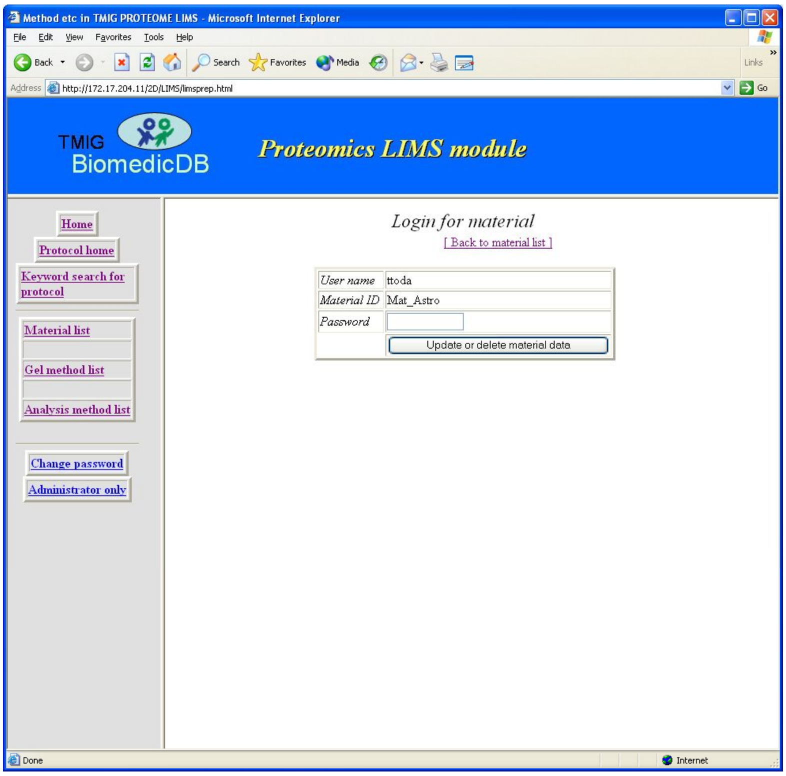
Method etc in TMIG PROTEOME LIMS - Microsoft Internet Explorer
http://172.17.204.11/2D/LIMS/limsprep.html
TMIG
BiomedicDB
Proteomics LIMS module
Home
Protocol home
Keyword search for protocol
Material list
Gel method list
Analysis method list
Change password
Administrator only
Login for material
[Back to material list]
User name
ttoda
Material ID
Mat_Astro
Password
Update or delete material data

Fig.15

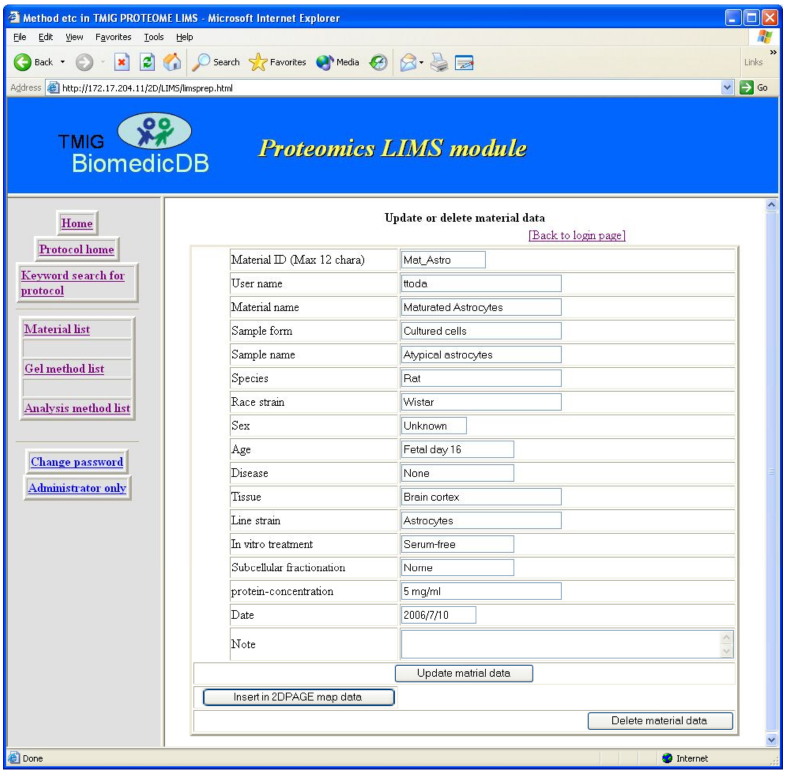
Method etc in TMIG PROTEOME LIMS - Microsoft Internet Explorer
http://172.17.204.11/2D/LIMS/limsprep.html
TMIG
BiomedicDB
Proteomics LIMS module
Home
Protocol home
Keyword search for protocol
Material list
Gel method list
Analysis method list
Change password
Administrator only
Update or delete material data
[Back to login page]
Material ID (Max 12 chara)
Mat_Astro
User name
ttoda
Material name
Maturated Astrocytes
Sample form
Cultured cells
Sample name
Atypical astrocytes
Species
Rat
Race strain
Wistar
Sex
Unknown
Age
Fetal day 16
Disease
None
Tissue
Brain cortex
Line strain
Astrocytes
In vitro treatment
Serum-free
Subcellular fractionation
Nome
protein-concentration
5 mg/ml
Date
2006/7/10
Note
Update matrial data
Insert in 2DPAGE map data
Delete material data

Fig.16

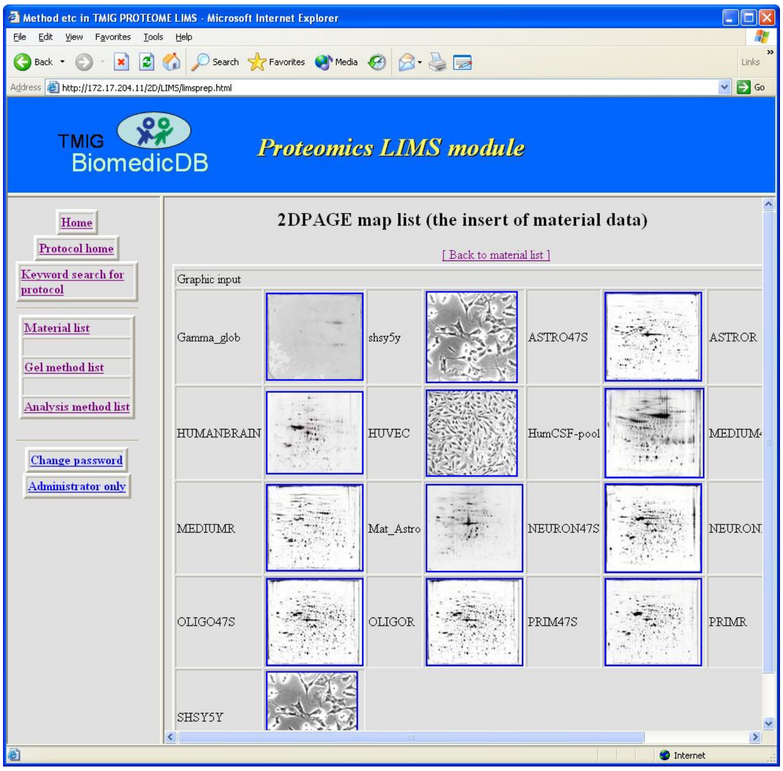
Method etc in TMIG PROTEOME LIMS - Microsoft Internet Explorer
File Edit View Favorites Tools Help
Back Search Favorites Media
Address http://172.17.204.11/2D/LIMS/limsprep.html
TMIG
BiomedicDB
Proteomics LIMS module
Home
Protocol home
Keyword search for protocol
Material list
Gel method list
Analysis method list
Change password
Administrator only
2DPAGE map list (the insert of material data)
[Back to material list]
Graphic input
Gamma_glob
shsy5y
ASTRO47S
ASTROR
HUMANBRAIN
HUVEC
HumCSF-pool
MEDIUM4
MEDIUMR
Mat_Astro
NEURON47S
NEURON
OLIGO47S
OLIGOR
PRIM47S
PRIMR
SHSY5Y
Internet

Fig.17

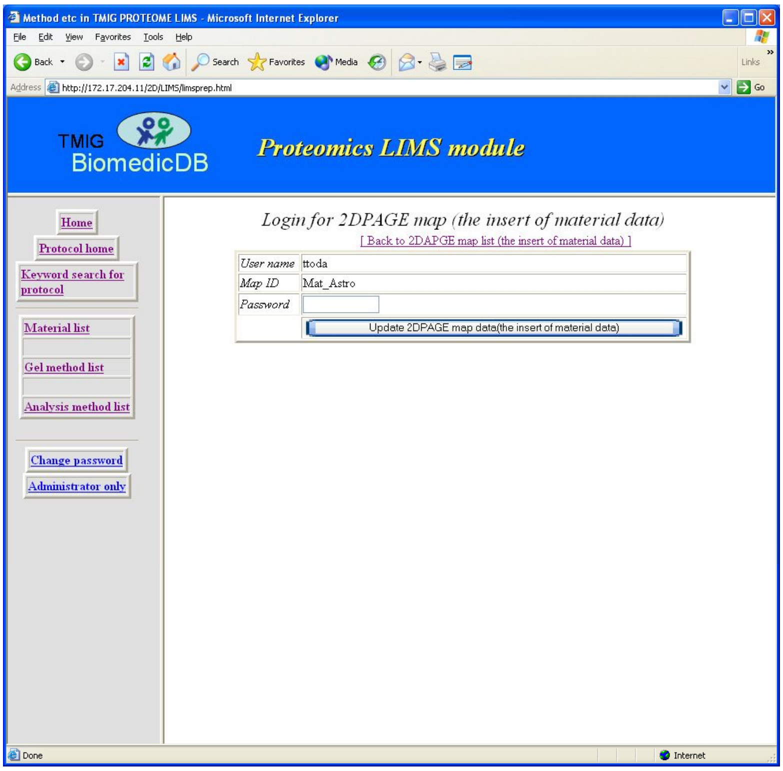
Method etc in TMIG PROTEOME LIMS - Microsoft Internet Explorer
File Edit View Favorites Tools Help
Back Search Favorites Media
Address http://172.17.204.11/2D/LIMS/limsprep.html
TMIG
BiomedicDB
Proteomics LIMS module
Home
Protocol home
Keyword search for protocol
Material list
Gel method list
Analysis method list
Change password
Administrator only
Login for 2DPAGE map (the insert of material data)
[Back to 2DAPGE map list (the insert of material data)]
User name ttoda
Map ID Mat_Astro
Password
Update 2DPAGE map data(the insert of material data)
Done
Internet

Fig.18

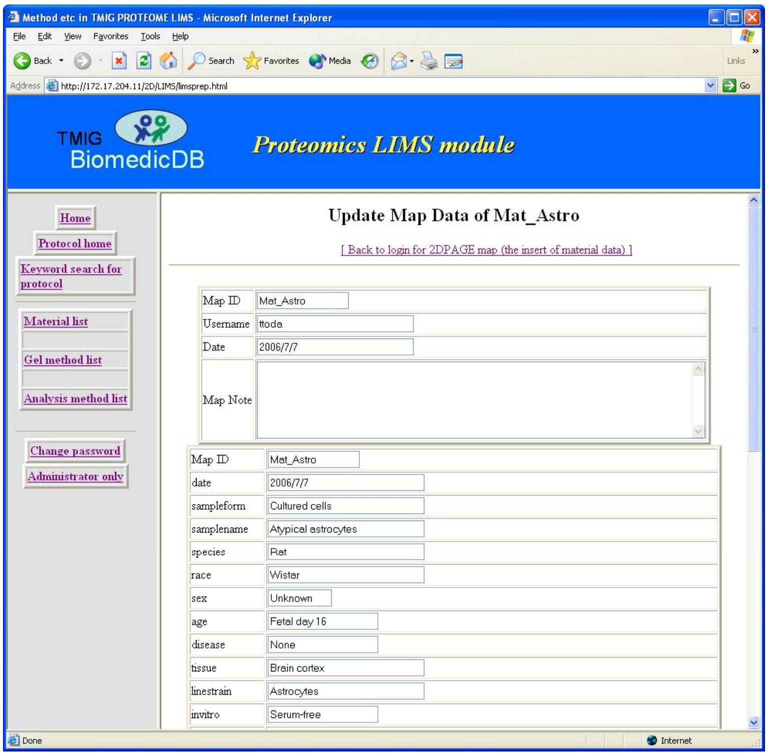

Fig.19

Secondly in the “Protocol home” window, enter the User name (ex. morisawa), Password (****) and Gel method ID (ex. mori_test) in each box, and click on “Go” button (Fig.12). Then the window of “Add gel method data of mori_test” appears. In this window, enter the each item, and click on “Add gel method data” button (Fig.20). Then the window of “The insert of data is OK!“ appears. In this window, click on “Gel method list” link button. Then the window of “Gel method list” appears.

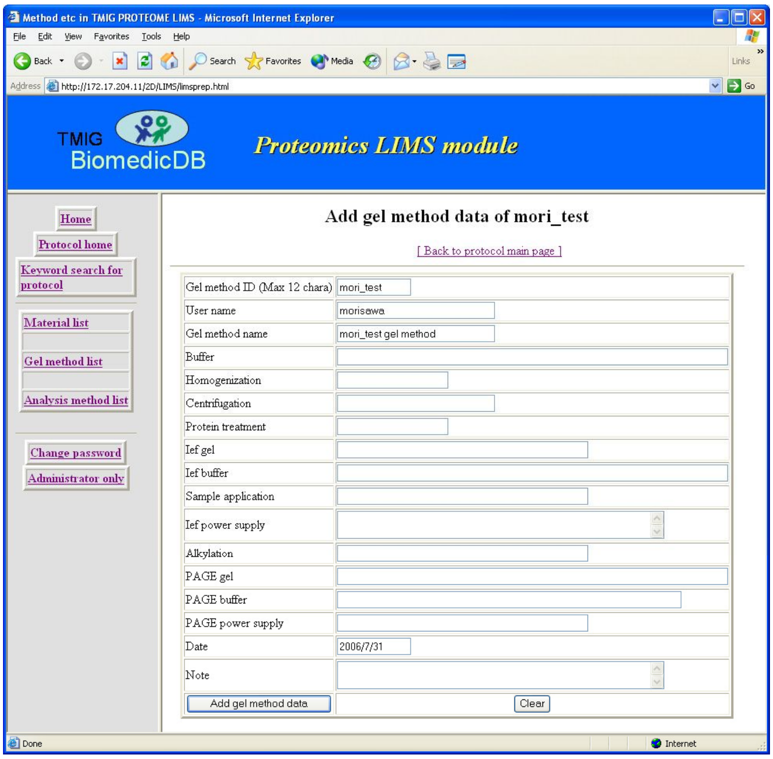

Fig.20

In the window, click on “Gel method ID (ex. Standard_2DE)” link button (Fig.21). Then

the window of “Login for gel method” appears. Enter the Password (****), and click on “Update or delete gel method data” button (Fig.22). Then the window of “Update or delete gel method data” appears. In this window, input experimental gel method data in each box, and update or delete gel method data(Fig.23). In addition, click on “Insert in 2DEPAGE map data” button, and then “2DPAGE map list” appears. In this window, click on the user’s gel icon (ex. Mat_Astro) (Fig.24). Then the window of “Login for 2DPAGE map (the insert of gel method data)” appears. Enter the Password (****), and click on “Update 2DPAGE map data (the insert of gel method data)” button (Fig.25). Then the window of “Update Map Data of Mat_Astro” appears. Input the data in each box, and click on “Update 2DPAGE map data” (Fig.26).

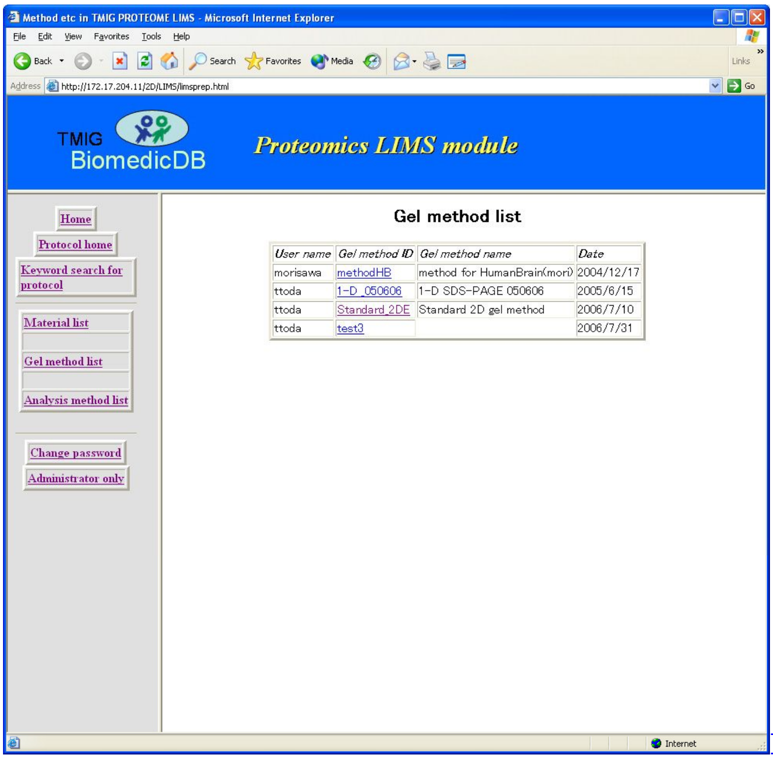

Fig.21

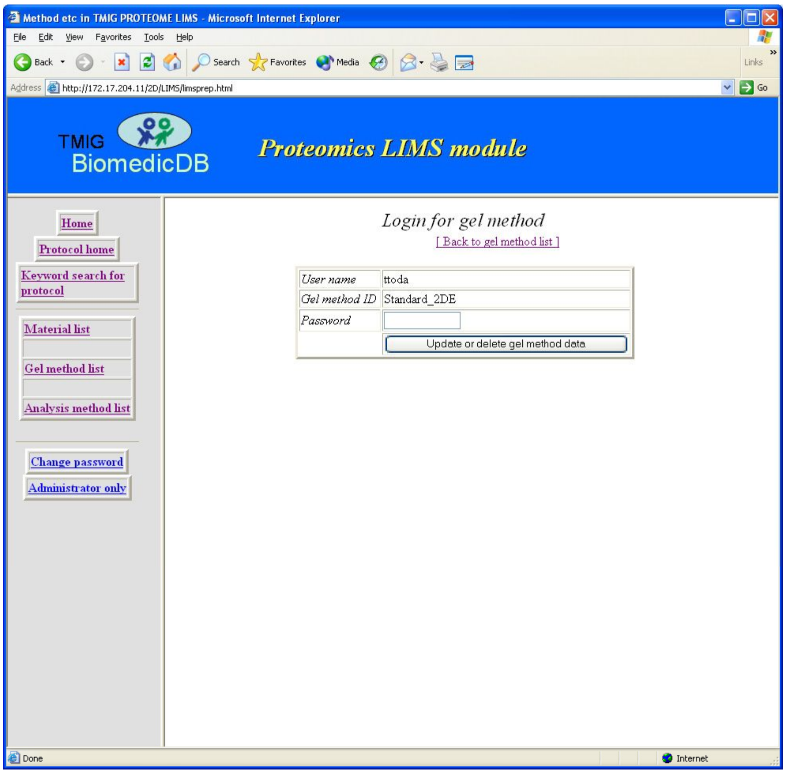
Method etc in TMIG PROTEOME LIMS - Microsoft Internet Explorer
http://172.17.204.11/2D/LIMS/limsprep.html
TMIG
BiomedicDB
Proteomics LIMS module
Home
Protocol home
Keyword search for protocol
Material list
Gel method list
Analysis method list
Change password
Administrator only
Login for gel method
[Back to gel method list]
User name
ttoda
Gel method ID
Standard_2DE
Password
Update or delete gel method data
Done
Internet

Fig.22

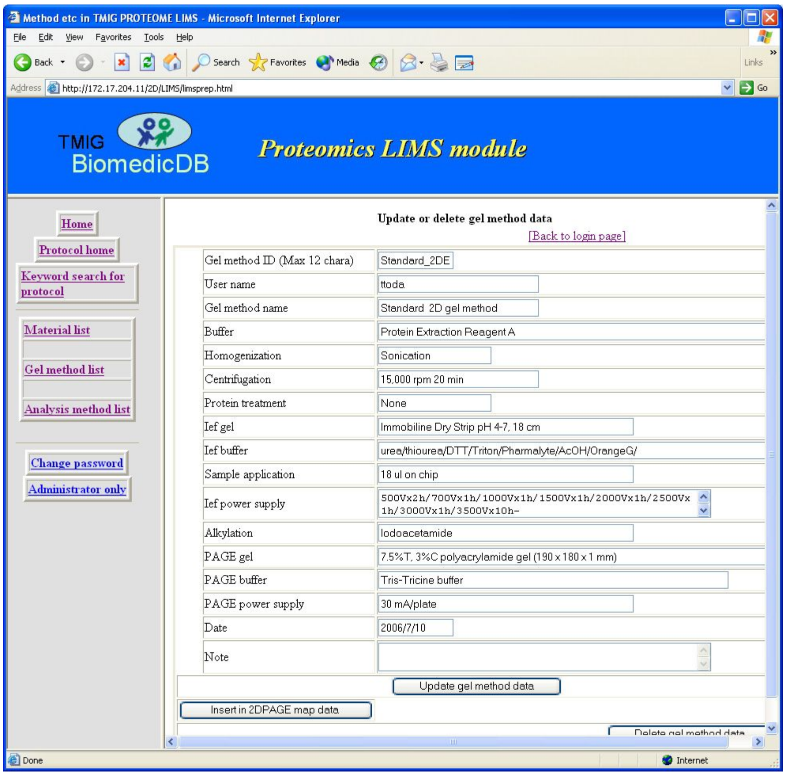
Method etc in TMIG PROTEOME LIMS - Microsoft Internet Explorer
http://172.17.204.11/2D/LIMS/limsprep.html
TMIG
BiomedicDB
Proteomics LIMS module
Home
Protocol home
Keyword search for protocol
Material list
Gel method list
Analysis method list
Change password
Administrator only
Update or delete gel method data
[Back to login page]
Gel method ID (Max 12 chara)
Standard_2DE
User name
ttoda
Gel method name
Standard 2D gel method
Buffer
Protein Extraction Reagent A
Homogenization
Sonication
Centrifugation
15,000 rpm 20 min
Protein treatment
None
Ief gel
Immobiline Dry Strip pH 4-7, 18 cm
Ief buffer
urea/thiourea/DTT/Triton/Pharmalyte/AcOH/OrangeG/
Sample application
18 ul on chip
Ief power supply
500Vx2h/700Vx1h/1000Vx1h/1500Vx1h/2000Vx1h/2500Vx
1h/3000Vx1h/3500Vx10h~
Alkylation
Iodoacetamide
PAGE gel
7.5%T, 3%C polyacrylamide gel (190 x 180 x 1 mm)
PAGE buffer
Tris-Tricine buffer
PAGE power supply
30 mA/plate
Date
2006/7/10
Note
Update gel method data
Insert in 2DPAGE map data
Done
Internet

Fig.23

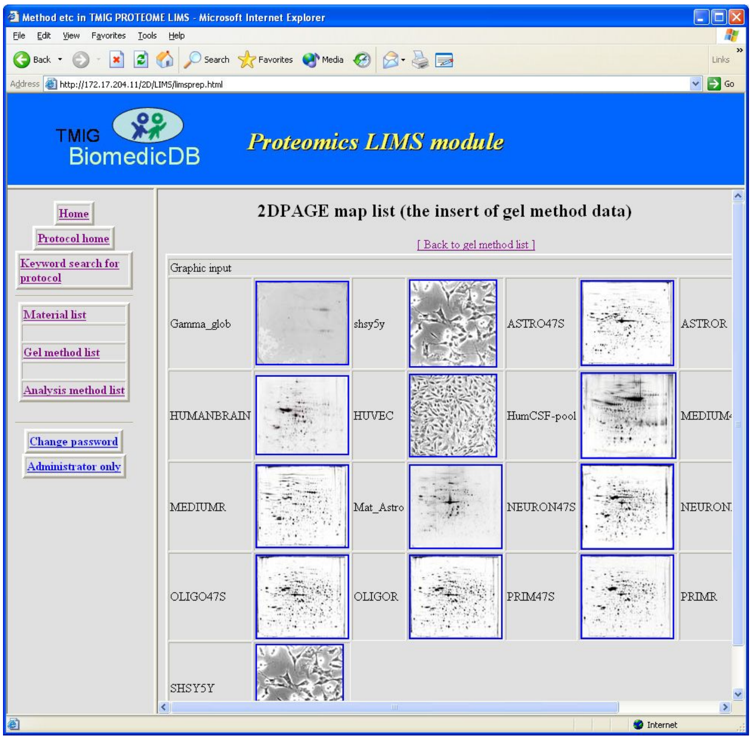
Method etc in TMIG PROTEOME LIMS - Microsoft Internet Explorer
TMIG BiomedicDB
Proteomics LIMS module
Home
Protocol home
Keyword search for protocol
Material list
Gel method list
Analysis method list
Change password
Administrator only
2DPAGE map list (the insert of gel method data)
[Back to gel method list]
Graphic input
Gamma_glob
shsy5y
ASTRO47S
ASTROR
HUMANBRAIN
HUVEC
HumCSF-pool
MEDIUMR
Mat_Astro
NEURON47S
OLIGO47S
OLIGOR
PRIM47S
PRIMR
SHSY5Y

Fig.24

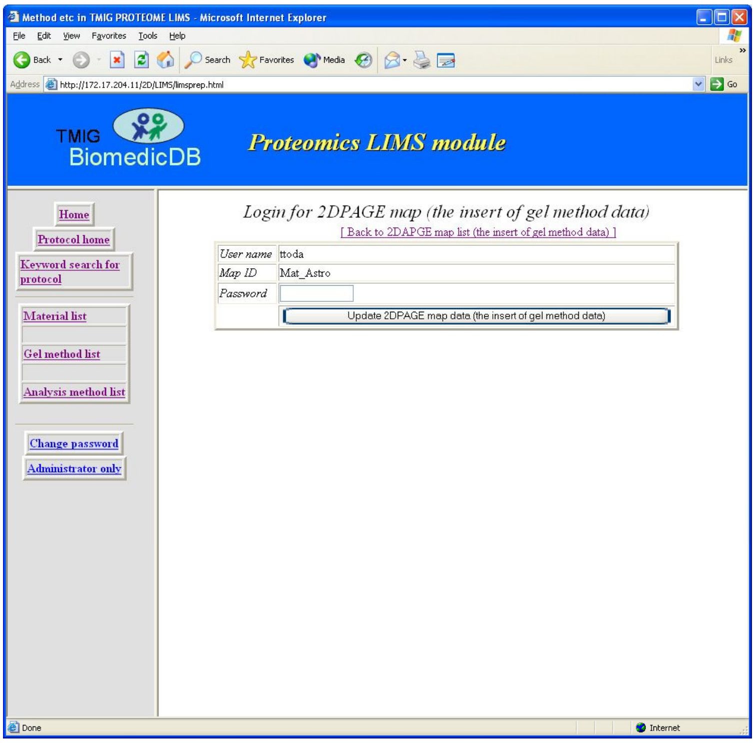
Method etc in TMIG PROTEOME LIMS - Microsoft Internet Explorer
TMIG BiomedicDB
Proteomics LIMS module
Home
Protocol home
Keyword search for protocol
Material list
Gel method list
Analysis method list
Change password
Administrator only
Login for 2DPAGE map (the insert of gel method data)
[Back to 2DAPGE map list (the insert of gel method data)]
User name ttoda
Map ID Mat_Astro
Password
Update 2DPAGE map data (the insert of gel method data)

Fig.25

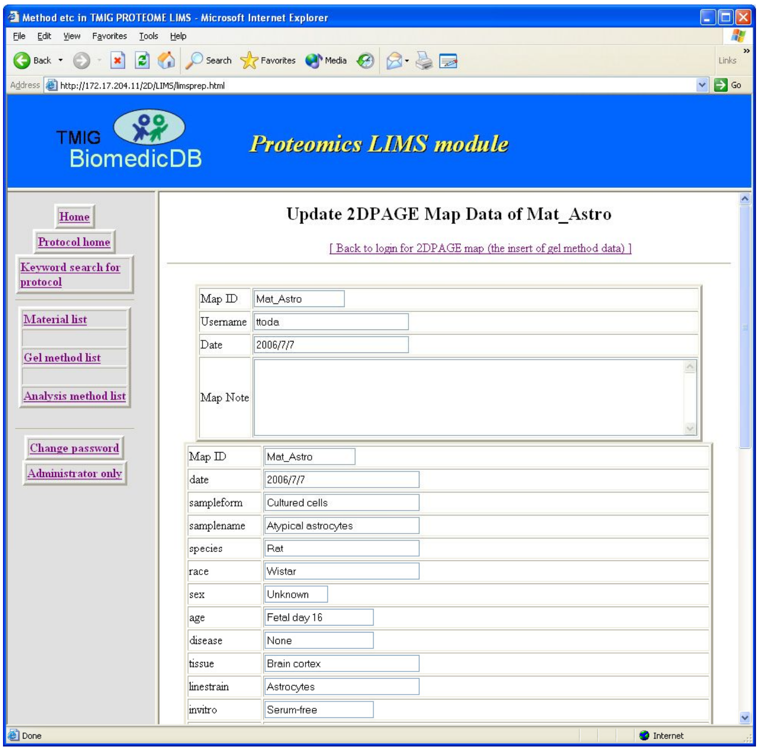

Fig.26

Finally in the “Protocol home” window, enter the User name (ex. morisawa), Password (****) and Analysis method ID (ex. mori_test) in each box, and click on “Go” button (Fig.12). Then the window of “Add analysis method data of mori_test” appears. In this window, enter the each item, and click on “Add analysis method data” button (Fig.27). Then the window of “The insert of data is OK!“ appears. In this window, click on “Analysis method list” link button. Then the window of “Analysis method list” appears.

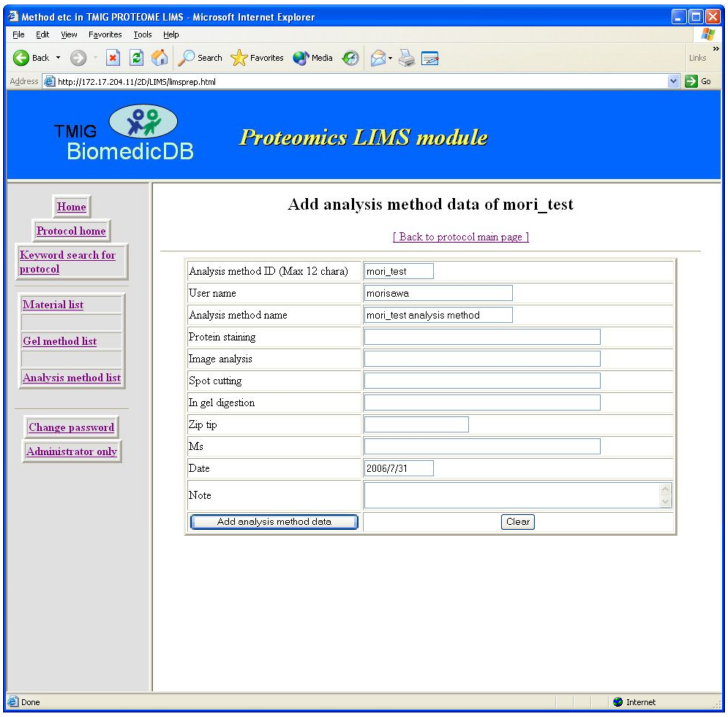

Fig.27

In the window, click on “Analysis method ID (ex. Toda_org)” link button (Fig.28). Then the window of “Login for analysis method” appears. Enter the Password (****), and click

on “Update or delete analysis method data” button (Fig.29). Then the window of “Update or delete analysis method data” appears. In this window, input experimental analysis method data in each box, and update or delete analysis method data(Fig.30). In addition, click on “Insert in 2DEPAGE map data” button, and then “2DPAGE map list” appears. In this window, click on the user’s gel icon (ex. Mat_Astro) (Fig.31). Then the window of “Login for 2DPAGE map (the insert of analysis method data)” appears. Enter the Password (****), and click on “Update 2DPAGE map data (the insert of analysis method data)” button (Fig.32). Then the window of “Update Map Data of Mat_Astro” appears. Input the data in each box, and click on “Update 2DPAGE map data” (Fig.33).

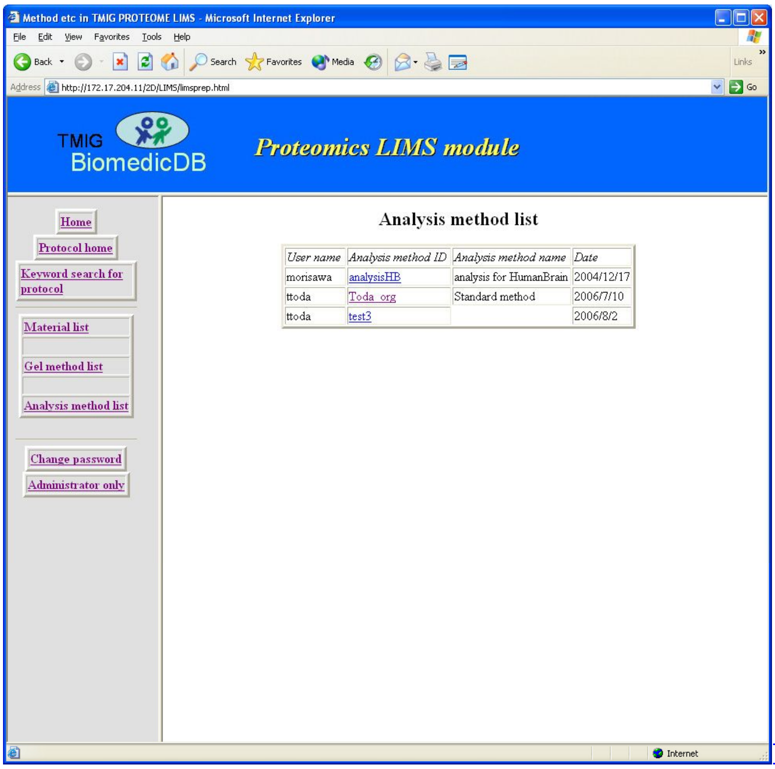

Fig.28

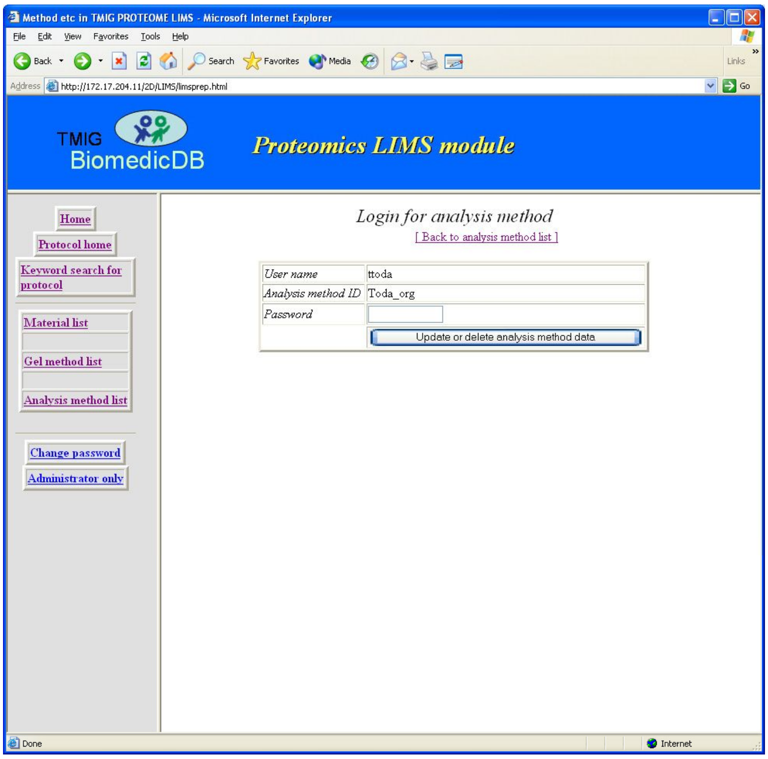
Method etc in TMIG PROTEOME LIMS - Microsoft Internet Explorer
http://172.17.204.11/2D/LIMS/limsprep.html
TMIG
BiomedicDB
Proteomics LIMS module
Home
Protocol home
Keyword search for protocol
Material list
Gel method list
Analysis method list
Change password
Administrator only
Login for analysis method
[Back to analysis method list]
User name
ttoda
Analysis method ID
Toda_org
Password
Update or delete analysis method data

Fig.29

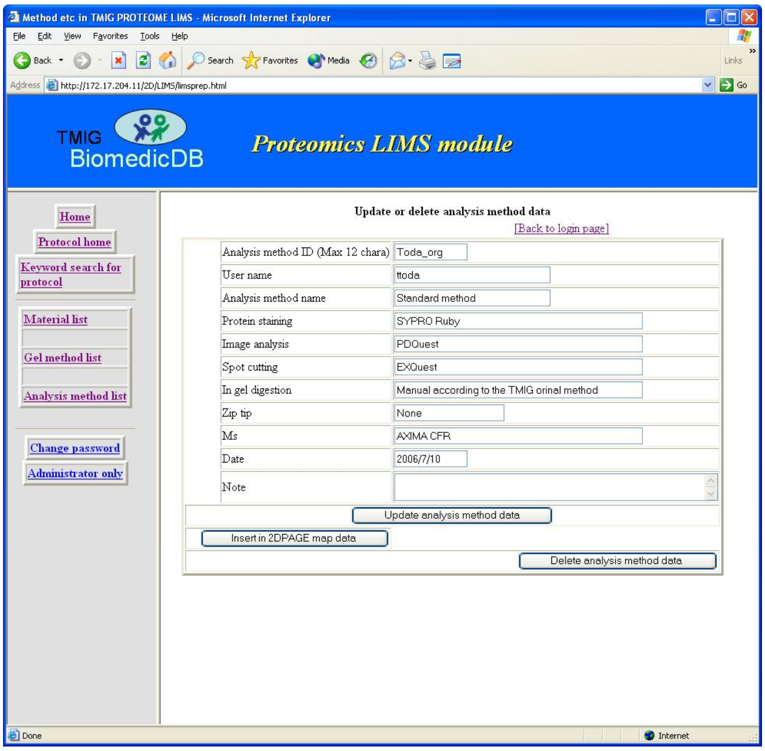
Method etc in TMIG PROTEOME LIMS - Microsoft Internet Explorer
http://172.17.204.11/2D/LIMS/limsprep.html
TMIG
BiomedicDB
Proteomics LIMS module
Home
Protocol home
Keyword search for protocol
Material list
Gel method list
Analysis method list
Change password
Administrator only
Update or delete analysis method data
[Back to login page]
Analysis method ID (Max 12 chara)
Toda_org
User name
ttoda
Analysis method name
Standard method
Protein staining
SYPRO Ruby
Image analysis
PDQuest
Spot cutting
EXQuest
In gel digestion
Manual according to the TMIG orinal method
Zip tip
None
Ms
AXIMA CFR
Date
2006/7/10
Note
Update analysis method data
Insert in 2DPAGE map data
Delete analysis method data

Fig.30

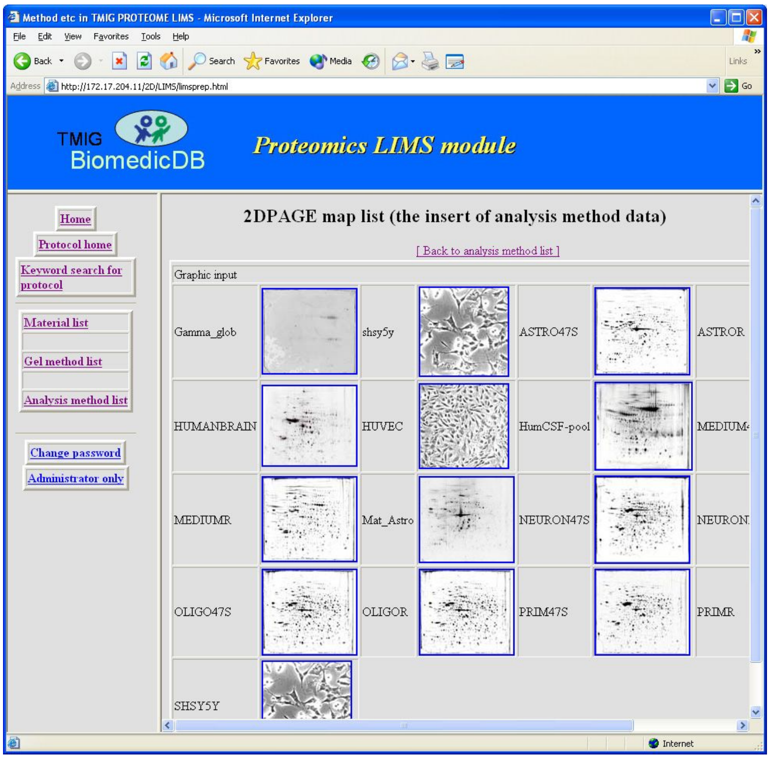
Method etc in TMIG PROTEOME LIMS - Microsoft Internet Explorer
http://172.17.204.11/2D/LIMS/limsprep.html
TMIG BiomedicDB
Proteomics LIMS module
Home
Protocol home
Keyword search for protocol
Material list
Gel method list
Analysis method list
Change password
Administrator only
2DPAGE map list (the insert of analysis method data)
[Back to analysis method list]
Graphic input
Gamma_glob
shsy5y
ASTRO47S
ASTROR
HUMANBRAIN
HUVEC
HumCSF-pool
MEDIUMR
Mat_Astro
NEURON47S
OLIGO47S
OLIGOR
PRIM47S
PRIMR
SHSY5Y

Fig.31

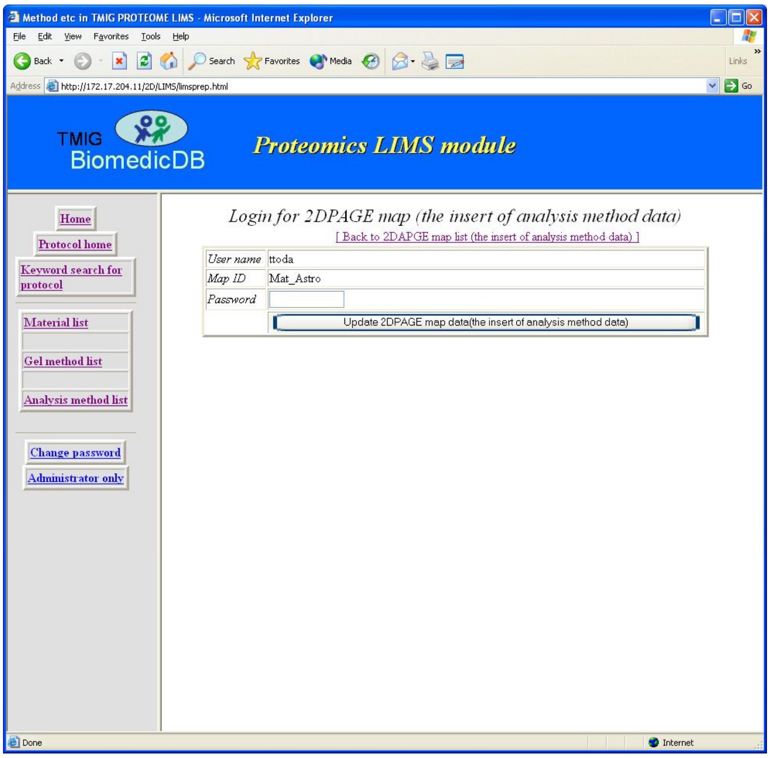
Method etc in TMIG PROTEOME LIMS - Microsoft Internet Explorer
http://172.17.204.11/2D/LIMS/limsprep.html
TMIG BiomedicDB
Proteomics LIMS module
Home
Protocol home
Keyword search for protocol
Material list
Gel method list
Analysis method list
Change password
Administrator only
Login for 2DPAGE map (the insert of analysis method data)
[Back to 2DAPGE map list (the insert of analysis method data)]
User name ttoda
Map ID Mat_Astro
Password
Update 2DPAGE map data(the insert of analysis method data)
Done

Fig.32

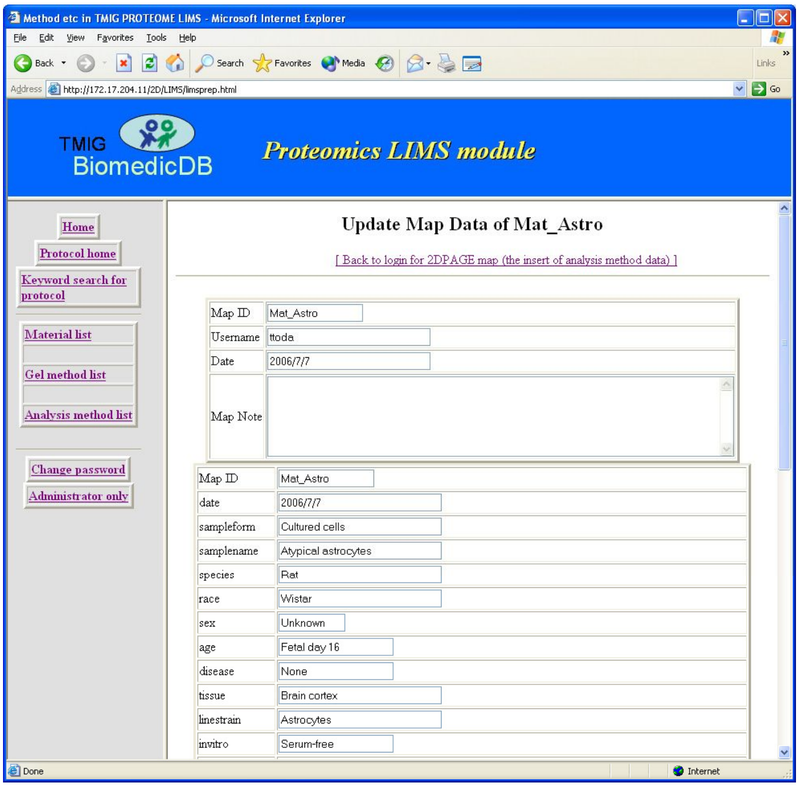

Method etc in TMIG PROTEOME LIMS - Microsoft Internet Explorer
http://172.17.204.11/2D/LIMS/limsprep.html
TMIG BiomedicDB
Proteomics LIMS module
Home
Protocol home
Keyword search for protocol
Material list
Gel method list
Analysis method list
Change password
Administrator only
Update Map Data of Mat_Astro
[Back to login for 2DPAGE map (the insert of analysis method data)]
Map ID Mat_Astro
Username ttoda
Date 2006/7/7
Map Note
Map ID Mat_Astro
date 2006/7/7
sampleform Cultured cells
samplename Atypical astrocytes
species Rat
race Wistar
sex Unknown
age Fetal day 16
disease None
tissue Brain cortex
linestrain Astrocytes
invitro Serum-free

Fig.33

Chapter 4 Component parts

4-1. Home

The window of “Home” including “Add or update 1&2DE-gel data”, “Add or update digestion plate data”, “Add or update MS plate data” and “Add or update 2DPAGE map data”.

Enter “User name”, “Password” and “ID number (1&2DE-gel ID, digestion plate, MS plate or 2DPAGE Map)”, and click on “Go” button.

4-2. Keyword search

The window of “Keyword search” including “Search 1 & 2DE-gels for spot data”, “Search digestion plates for well data”, “Search MS plates for well data”, “Search 2DPAGE database for spot data” and “Search 2DPAGE database for spot data (graph of protein expression)”.

Search by terms. Enter the term in each box, and click on “Search” button (Fig.34).

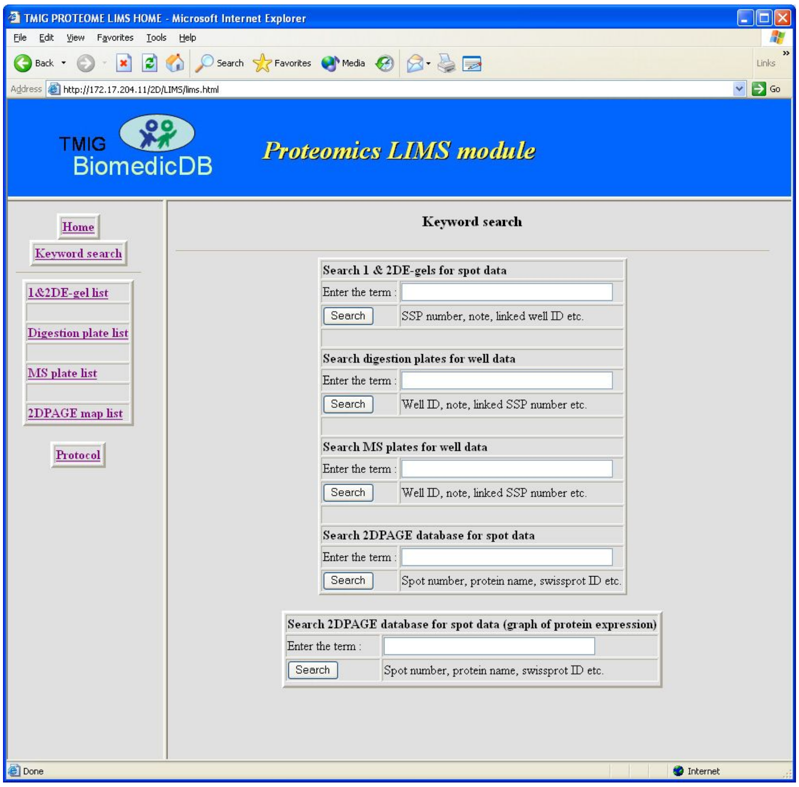

Fig.34

4-3. Editing workflow data

4-3-1.The window of “1&2DE-gel list” including “User name”, “1&2DE-gel ID”, 1&2DE-gel name”, “Date” and “Image” (Fig.35). If you select the 1&2DE-gel ID such as “Mat_Astro”, the window of “Login for 1&2DE-gel” will appear (Fig.36).

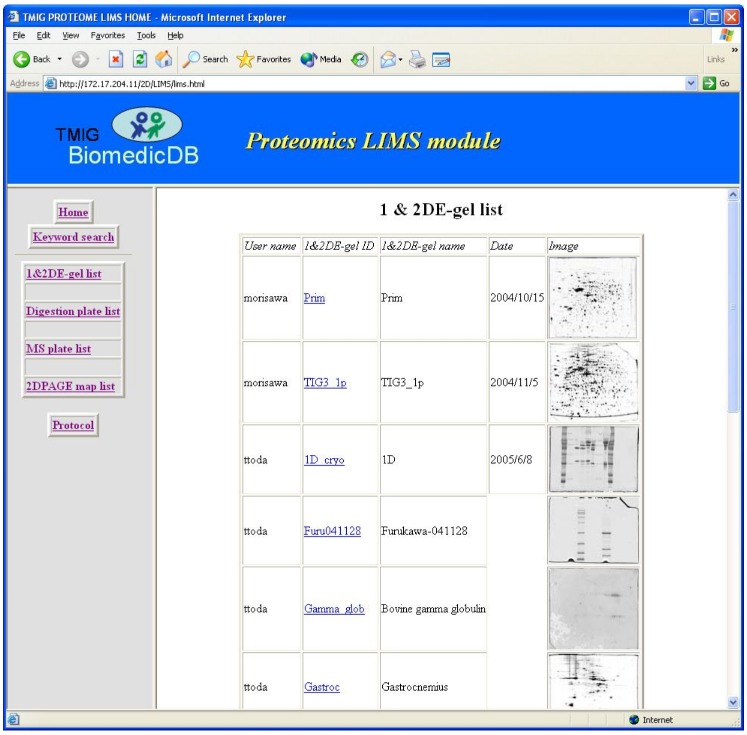

Fig.35

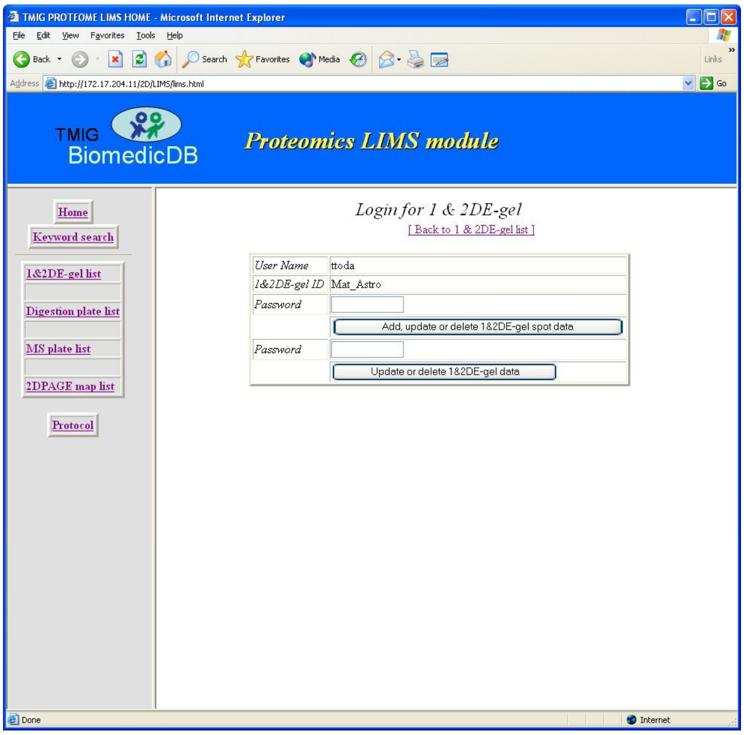

Fig.36

Enter the Password for "Add, update or delete 1&2DE-gel spot data", and then the window of "1&2DE-gel map of: Mat_Astro" appears (Fig.37). When you want to make the new position on this window, detect the spot position by moving a pointing mouse cursor, and click on "Detect the spot position" button. Rectangular width can be changed in accordance with the size of the spot. Then the window of "Add spot data in 1&2DE-gel" appears, and input the data in each box (Fig.38). If you select the SSP number such as "2201" by moving a pointing mouse cursor, the window of "Update or delete spot data of 1&2DE-gel" will appear (Fig.39).

Enter the Password for "Update or delete 1&2DE-gel data" (Fig.36). Then the window of "Update or delete 1&2DE-gel data of Mat_Astro" appears.

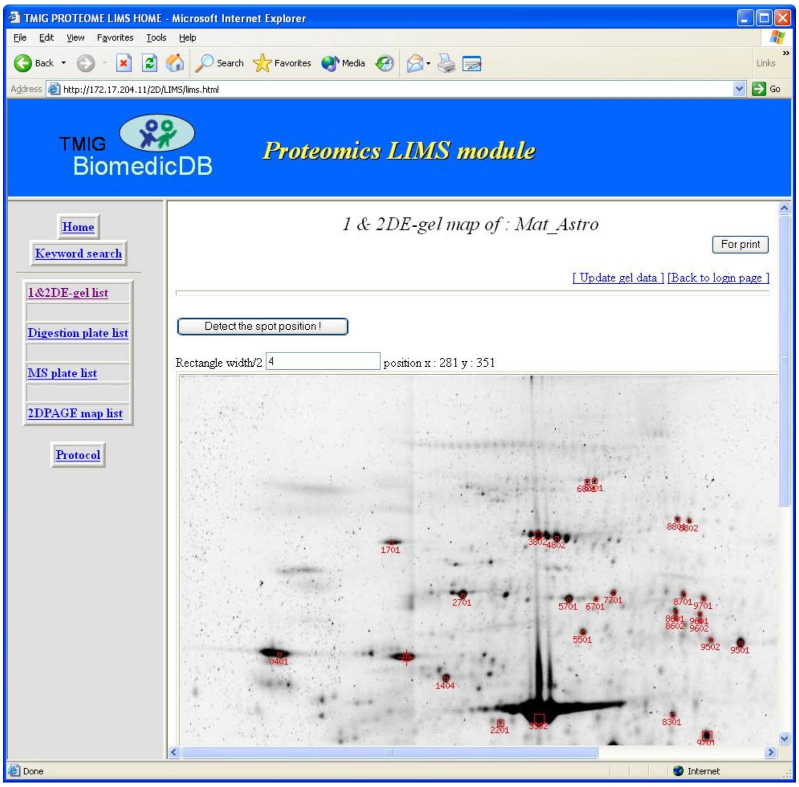
TMIG PROTEOME LIMS HOME - Microsoft Internet Explorer
http://172.17.204.11/2D/LIMS/lims.html
TMIG
BiomedicDB
Proteomics LIMS module
Home
Keyword search
1&2DE-gel list
Digestion plate list
MS plate list
2DPAGE map list
Protocol
1 & 2DE-gel map of : Mat_Astro
For print
[Update gel data] [Back to login page]
Detect the spot position !
Rectangle width/2
position x : 281 y : 351
Done
Internet

Fig.37

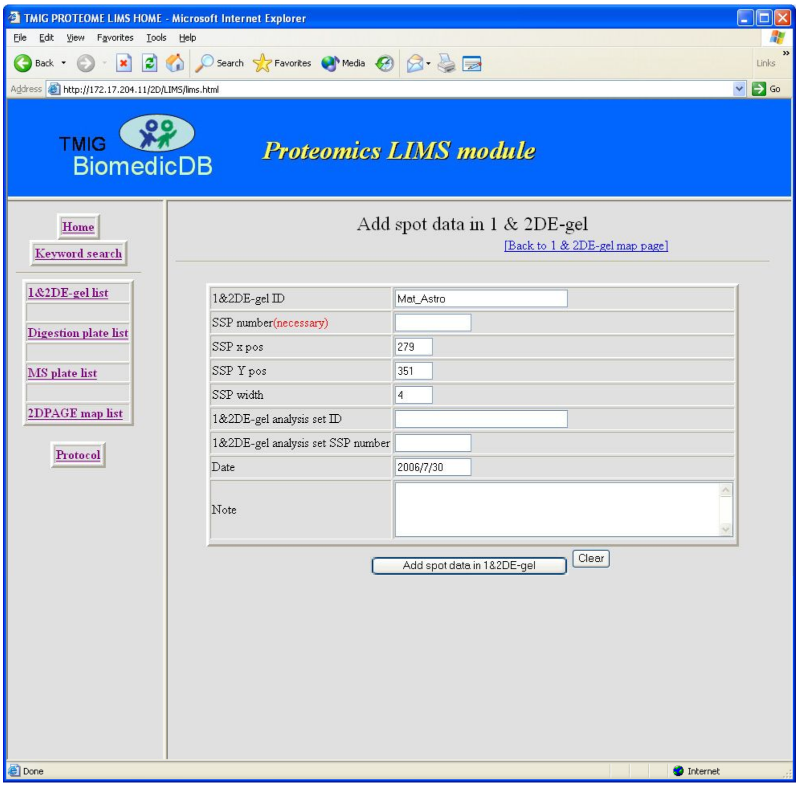
TMIG PROTEOME LIMS HOME - Microsoft Internet Explorer
http://172.17.204.11/2D/LIMS/lims.html
TMIG
BiomedicDB
Proteomics LIMS module
Home
Keyword search
1&2DE-gel list
Digestion plate list
MS plate list
2DPAGE map list
Protocol
Add spot data in 1 & 2DE-gel
[Back to 1 & 2DE-gel map page]
1&2DE-gel ID
Mat_Astro
SSP number(necessary)
SSP x pos
279
SSP Y pos
351
SSP width
4
1&2DE-gel analysis set ID
1&2DE-gel analysis set SSP number
Date
2006/7/30
Note
Add spot data in 1&2DE-gel
Clear
Done
Internet

Fig.38

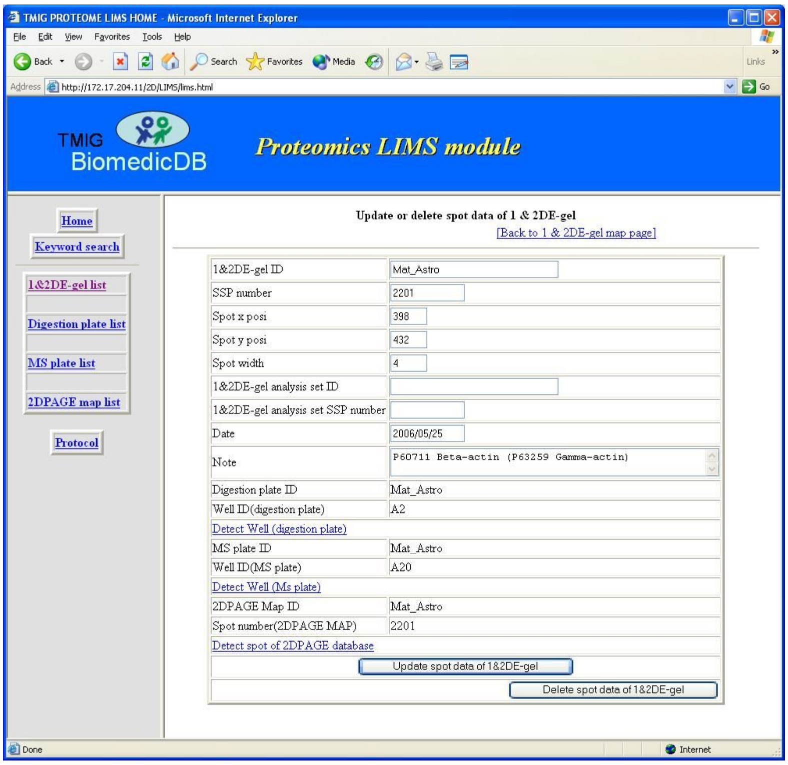

Fig.39

4-3-2. The window of "Digestion plate list" including "User name", "Digestion plate ID", "Digestion plate name" and "Date" (Fig.40). If you select the Digestion plate ID such as "Mat_Astro", the window of "Login for digestion plate " will appear (Fig.41).

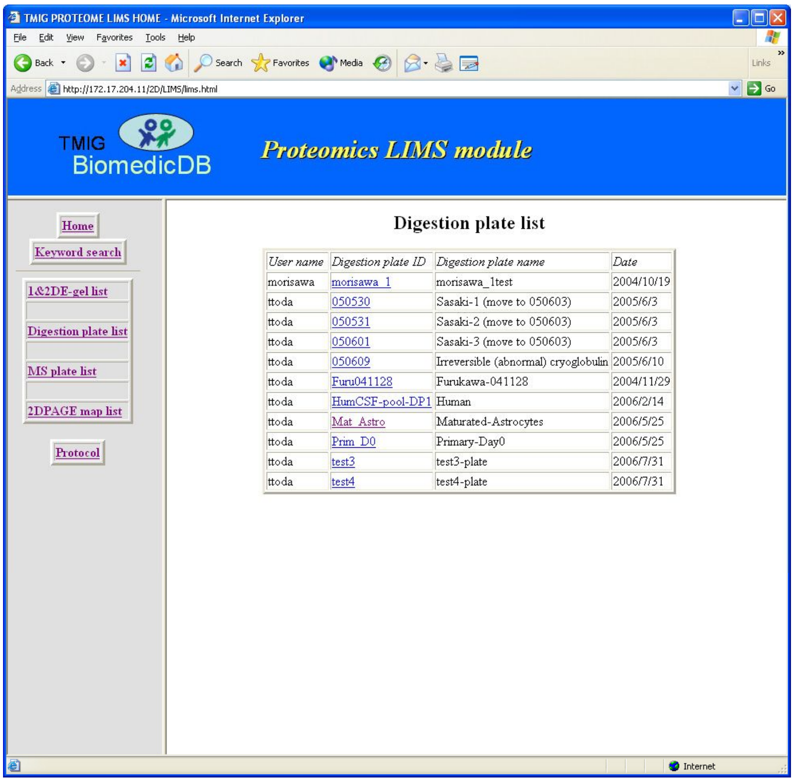

Fig.40

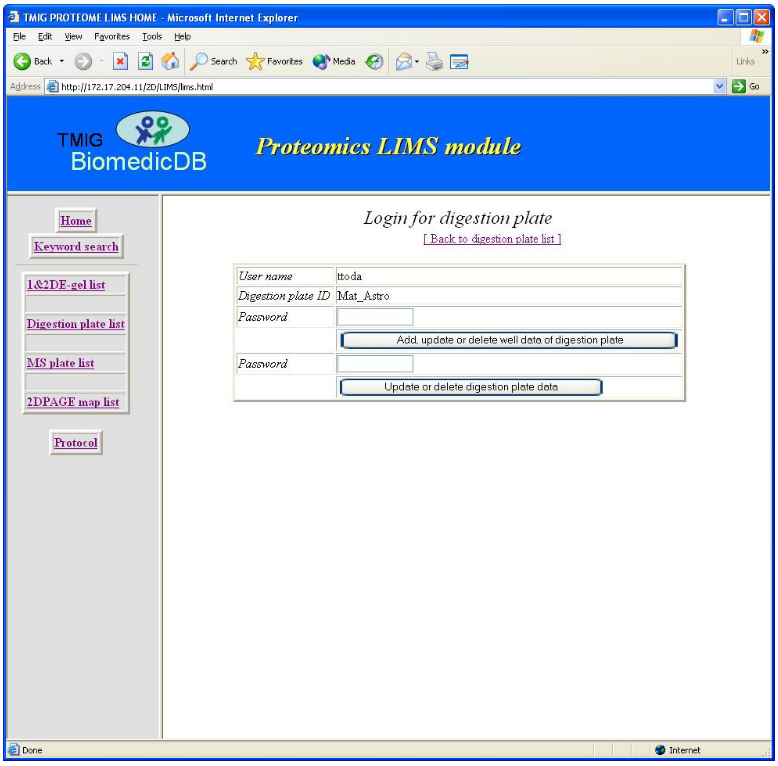

Fig.41

Enter the Password for "Add, update or delete well data of digestion plate", and then the window of "Digestion plate map of: Mat_Astro" appears (Fig.42). When you want to make the new position on this window, detect the well position by moving a pointing mouse cursor, and click on "Detect the well position" button. Then the window of "Add well data of digestion plate" appears, and enter the corresponding 1&2DE-gel ID and SSP number (Fig.43). If you select a Well ID such as "D5" by moving a pointing mouse cursor, the window of "Update or delete well data of digestion plate" will appear (Fig.44).

Enter the Password for "Update or delete digestion plate data" (Fig.41). Then the window of "Update or delete digestion data of Mat_Astro" appears.

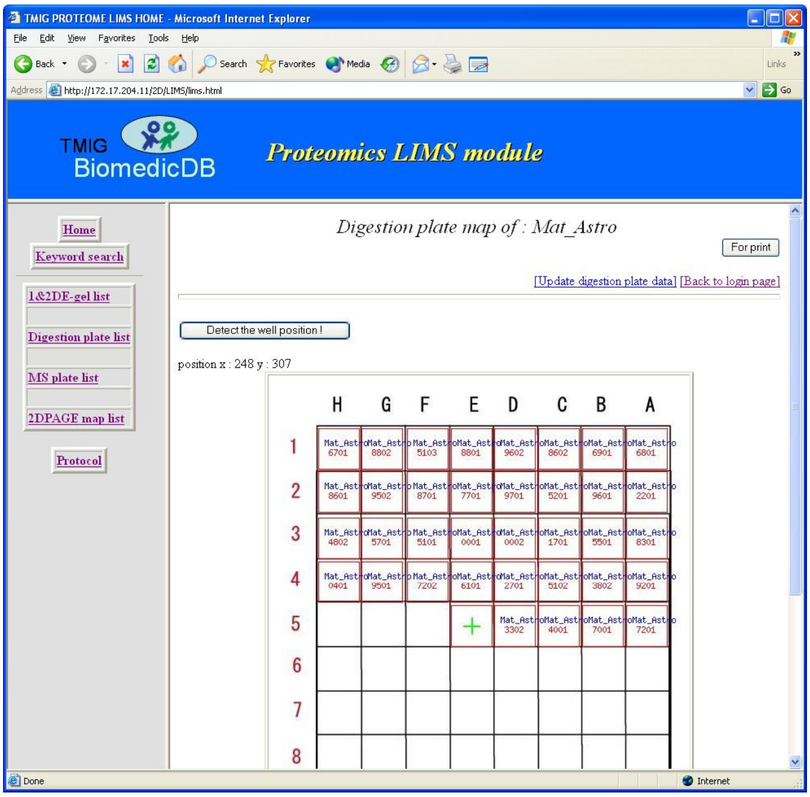
TMIG PROTEOME LIMS HOME - Microsoft Internet Explorer
http://172.17.204.11/2D/LIMS/lims.html
TMIG BiomedicDB
Proteomics LIMS module
Home
Keyword search
1&2DE-gel list
Digestion plate list
MS plate list
2DPAGE map list
Protocol
Digestion plate map of : Mat_Astro
For print
[Update digestion plate data] [Back to login page]
Detect the well position !
position x : 248 y : 307
H G F E D C B A
1 2 3 4 5 6 7 8
Done
Internet

Fig.42

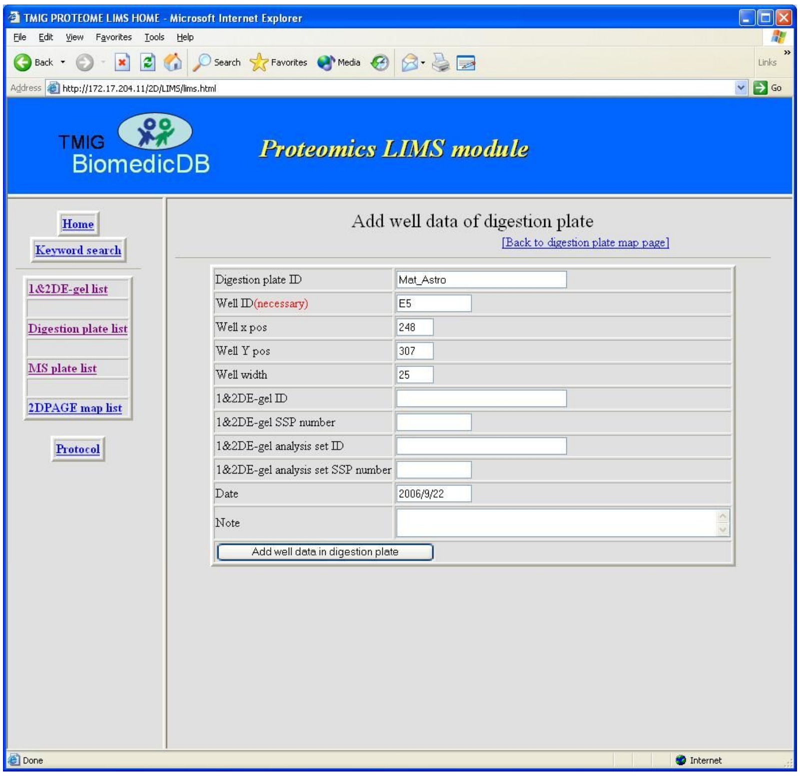
TMIG PROTEOME LIMS HOME - Microsoft Internet Explorer
http://172.17.204.11/2D/LIMS/lims.html
TMIG BiomedicDB
Proteomics LIMS module
Home
Keyword search
1&2DE-gel list
Digestion plate list
MS plate list
2DPAGE map list
Protocol
Add well data of digestion plate
[Back to digestion plate map page]
Digestion plate ID
Mat_Astro
Well ID(necessary)
E5
Well x pos
248
Well Y pos
307
Well width
25
1&2DE-gel ID
1&2DE-gel SSP number
1&2DE-gel analysis set ID
1&2DE-gel analysis set SSP number
Date
2006/9/22
Note
Add well data in digestion plate
Done
Internet

Fig.43

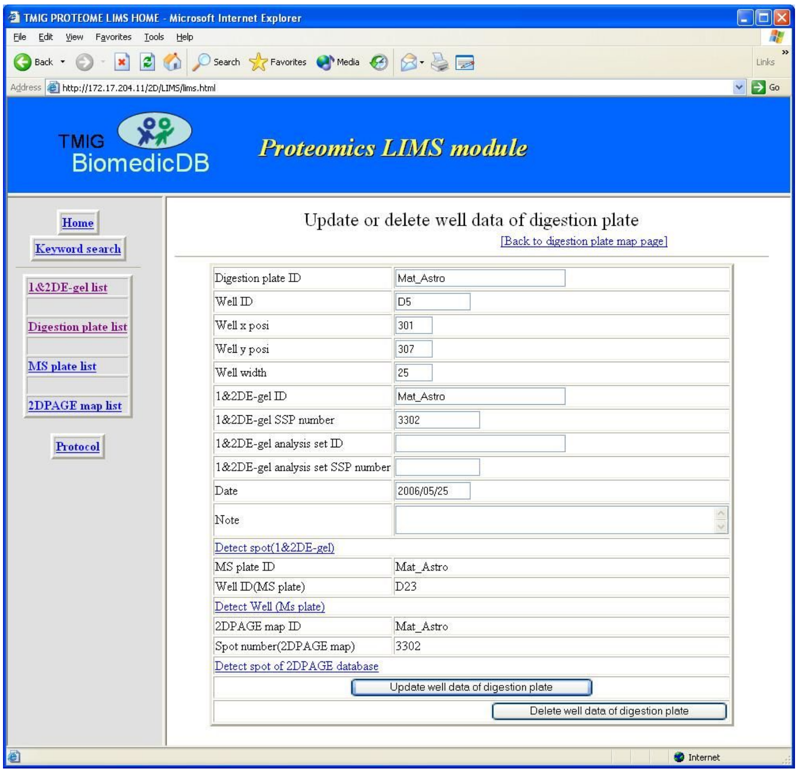

Fig.44

4-3-3. The window of “MS plate list” including “User name”, “MS plate ID”, “MS plate name” and “Date” (Fig.45). If you select the MS plate ID such as “Mat_Astro”, the window of “Login for MS plate “ will appear (Fig.46).

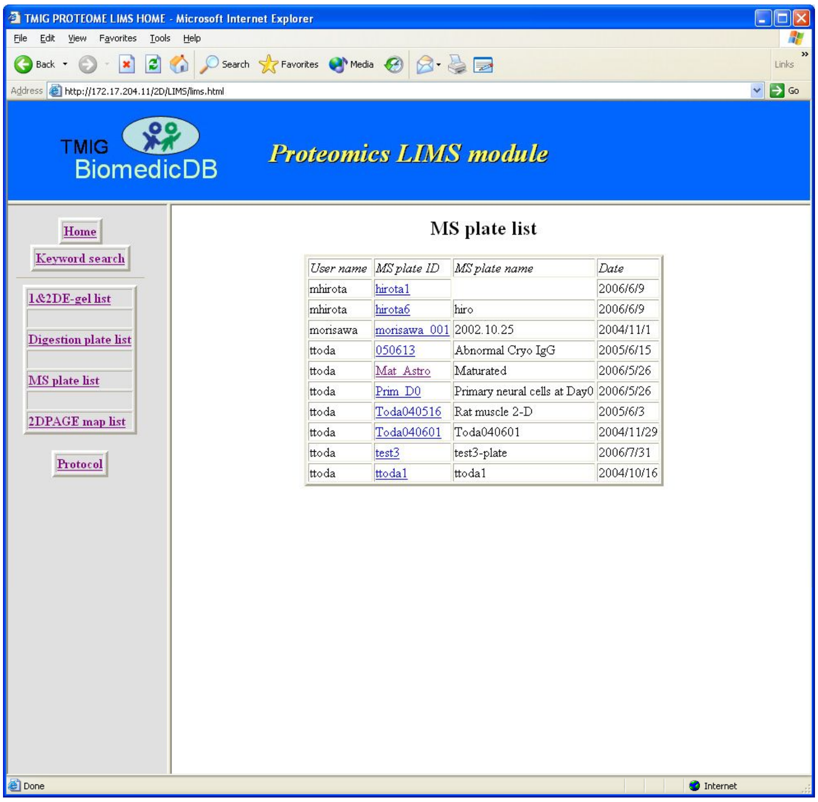

Fig.45

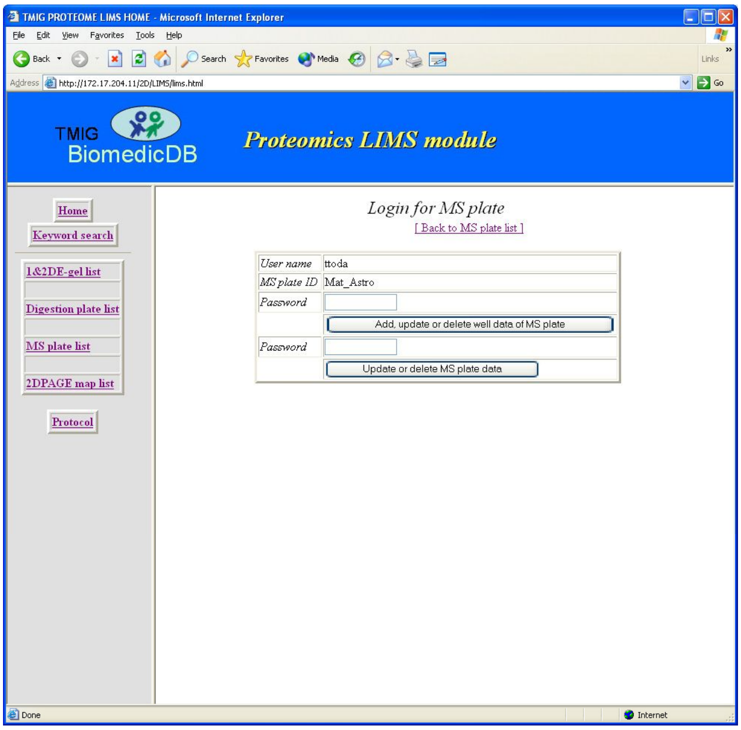

Fig.46

Enter the Password for “Add, update or delete well data of MS plate”, and then the window of “MS plate map of Mat_Astro” appears (Fig.47). When you want to make the new position on this window, detect the well position by moving a pointing mouse cursor, and click on “Detect the well position” button. Then the window of “Add well data of MS plate” appears, and enter the corresponding digestion plate ID and well ID (Fig.48). If you select the Well ID such as “D23” by moving a pointing mouse cursor, the window of “Update or delete well data of MS plate” will appear (Fig.49).

Enter the Password for “Update or delete MS plate data” (Fig.41). Then the window of “Update or delete MS plate data of Mat_Astro” appears.

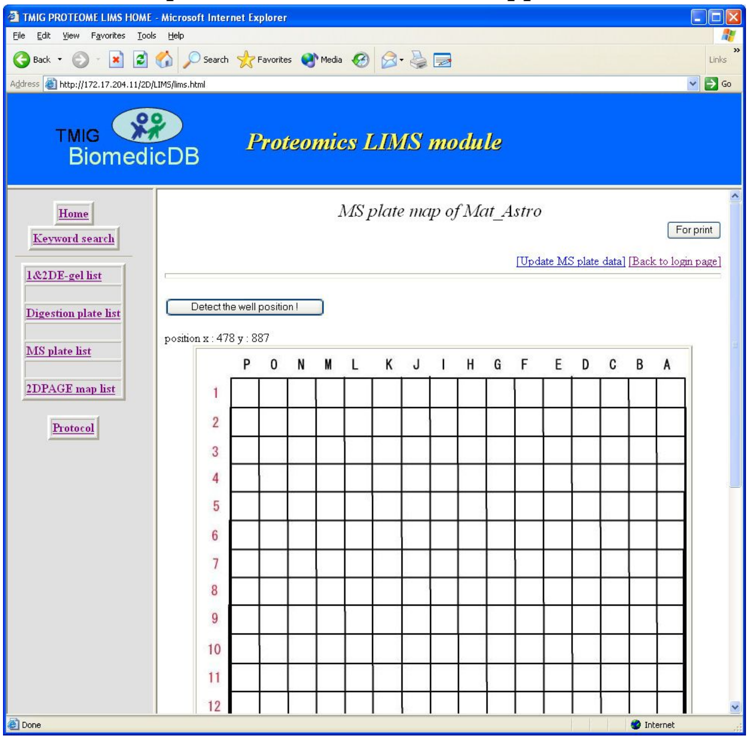

Fig.47

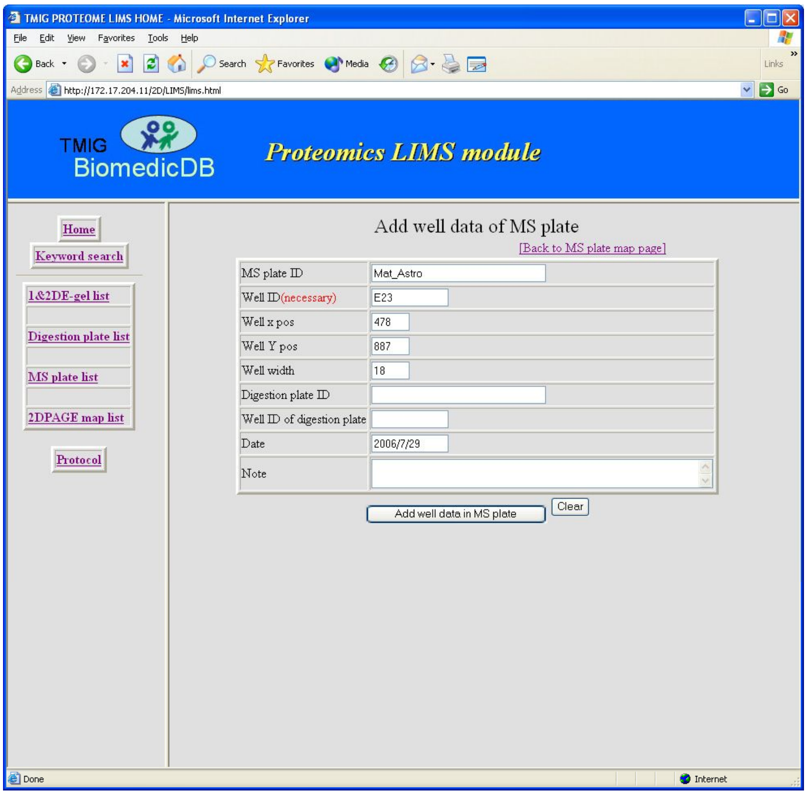

Fig.48

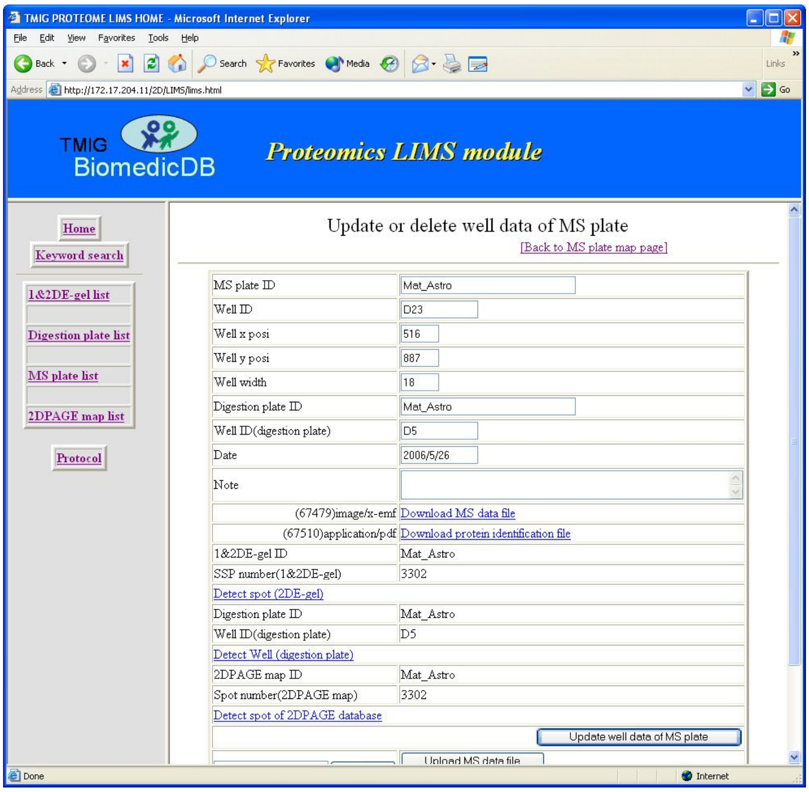

Fig.49

4-3-4. The window of "2D-PAGE map list" (Fig.50). If you select 2DPAGE map such as "Mat_Astro", the window of "Login for 2DPAGE map" will appear.

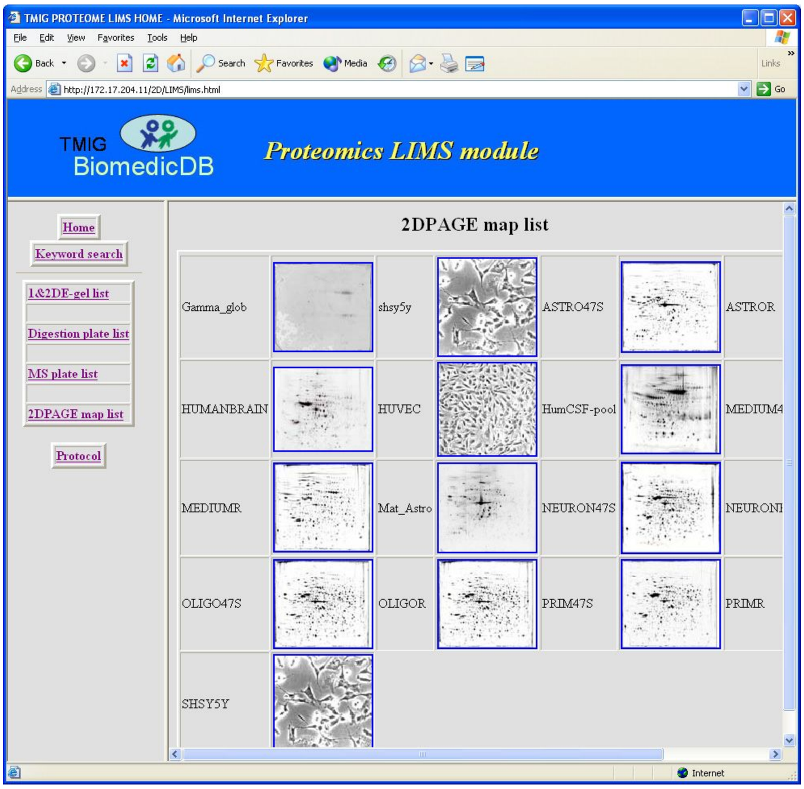

Fig.50

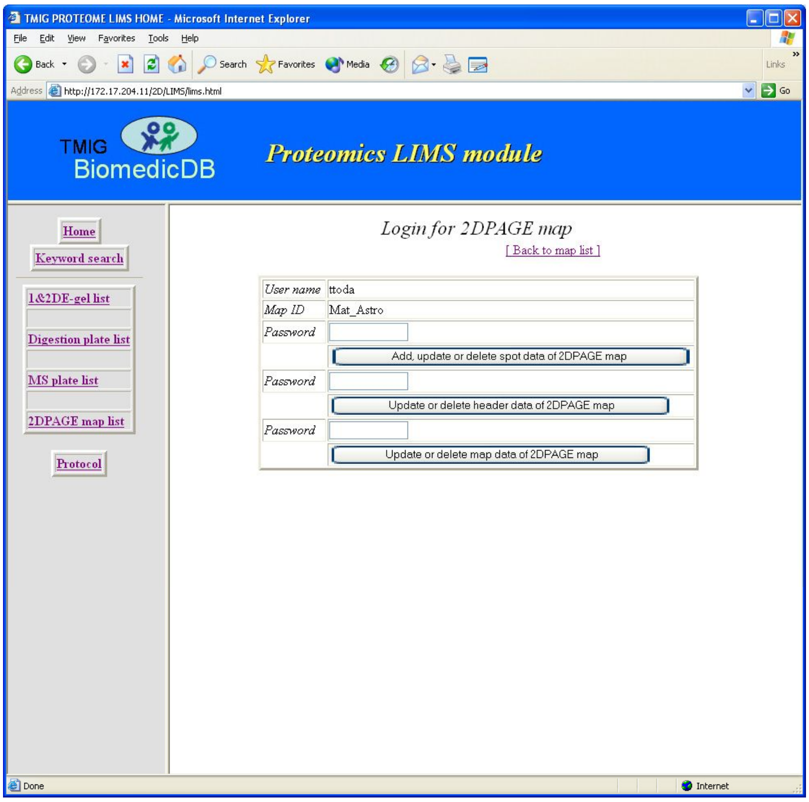

Fig.51

Enter the Password for "Add, update or delete spot data of 2DPAGE map" (Fig.51). Then the window of "2DPAGE map : Mat_Astro" appears (Fig.52). In this window, click on "Disp spot number" link button, the spot numbers appear. When you want to make the new position on this window, detect the spot position by moving a pointing mouse cursor, and click on "Detect the spot position" button. Rectangular width can be changed in accordance with the size of the spot. Then the window of "Add spot data in 2DPAGE

map" appears, and enter the corresponding MS plate ID, well ID and other data in each box (Fig.53). If you select the spot number such as "2201" by moving a pointing mouse cursor, the window of "Update or delete spot data of 2DPAGE map" will appear (Fig.54). When you click on "Detect Well (digestion plate)" link button, the window of "Well data in digestion plate" appears (Fig.55). Cross mark shows the well of "A2".

Enter the Password for "Update or delete header data of 2DPAGE map" or "Update map data of 2DPAGE map" (Fig.51). Then the window of "Update header data of 2DPAGE map" or "Update Map data of Mat_Astro" appears.

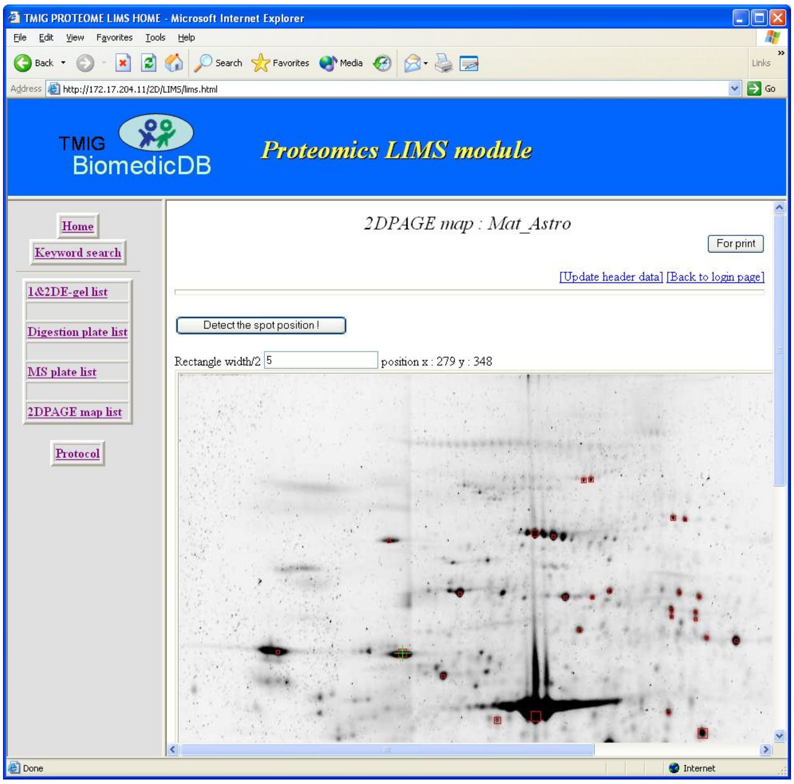

Fig.52

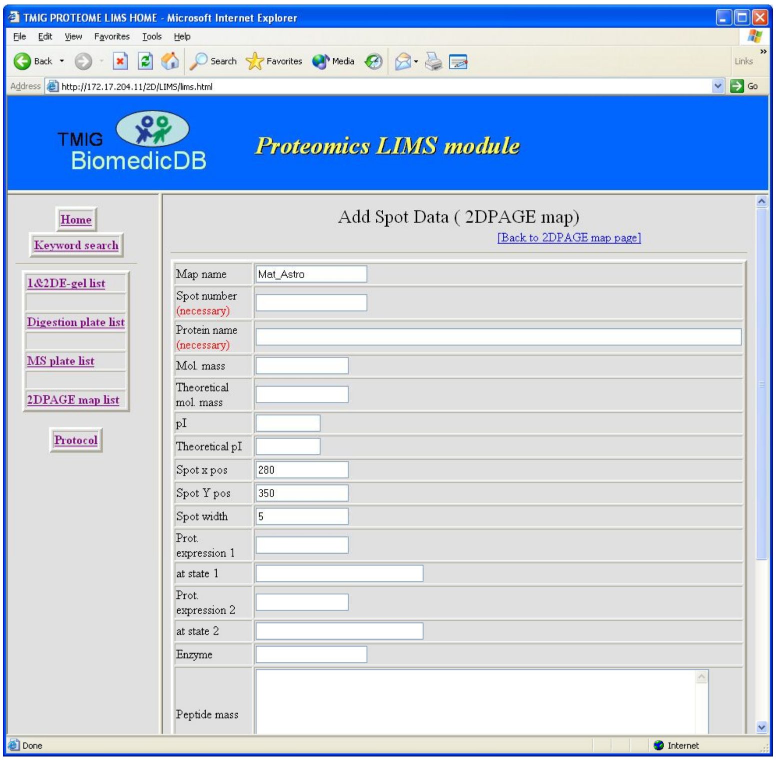
TMIG PROTEOME LIMS HOME - Microsoft Internet Explorer
http://172.17.204.11/2D/LIMS/lims.html
TMIG BiomedicDB
Proteomics LIMS module
Home
Keyword search
1&2DE-gel list
Digestion plate list
MS plate list
2DPAGE map list
Protocol
Add Spot Data (2DPAGE map)
[Back to 2DPAGE map page]
Map name
Mat_Astro
Spot number (necessary)
Protein name (necessary)
Mol. mass
Theoretical mol. mass
pI
Theoretical pI
Spot x pos
280
Spot Y pos
350
Spot width
5
Prot. expression 1
at state 1
Prot. expression 2
at state 2
Enzyme
Peptide mass

Fig.53

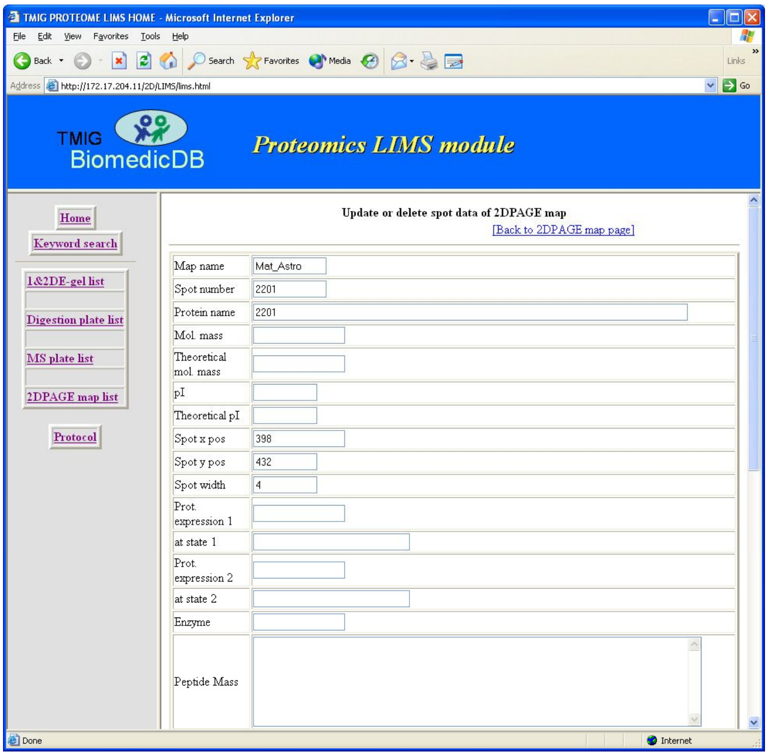
TMIG PROTEOME LIMS HOME - Microsoft Internet Explorer
http://172.17.204.11/2D/LIMS/lims.html
TMIG BiomedicDB
Proteomics LIMS module
Home
Keyword search
1&2DE-gel list
Digestion plate list
MS plate list
2DPAGE map list
Protocol
Update or delete spot data of 2DPAGE map
[Back to 2DPAGE map page]
Map name
Mat_Astro
Spot number
2201
Protein name
2201
Mol. mass
Theoretical mol. mass
pI
Theoretical pI
Spot x pos
398
Spot y pos
432
Spot width
4
Prot. expression 1
at state 1
Prot. expression 2
at state 2
Enzyme
Peptide Mass

Fig.54

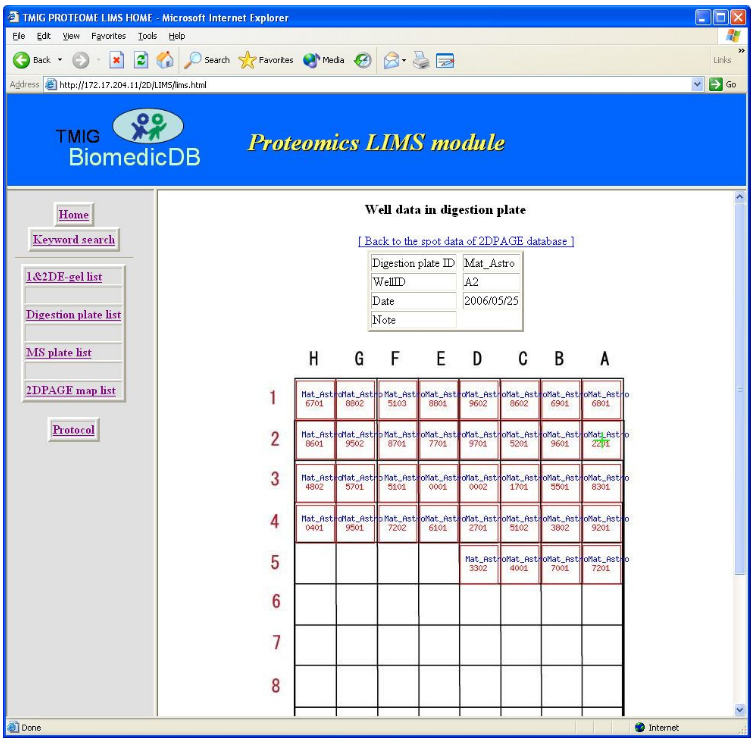

Fig.55

4-4. Protocol data

4-4-1. The window of "Protocol Home" including "Add or update material data", "Add or update gel method data" and "Add or update analysis method data".

Enter "User name", "Password" and "ID number (material ID, method ID or analysis ID) ", and then click on "Go" button (Fig.12).

4-4-2. The window of "Keyword search for protocol" including "Search material data by keyword", "Search gel method data by keyword" and "Search analysis method by keyword"

Search by terms. Enter the term in each box, and click on "Search" button (Fig.56).

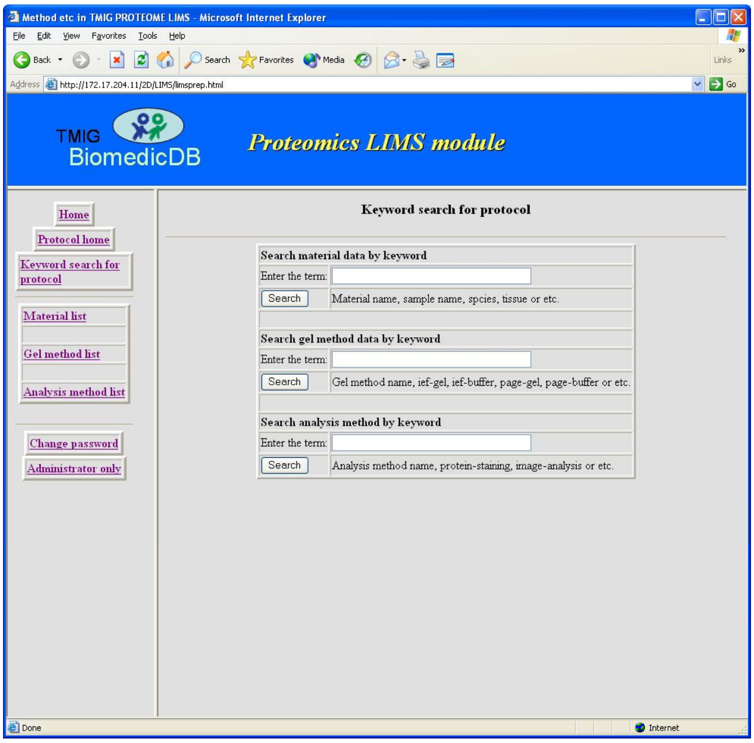

Fig.56

4-4-3. The window of "Material list" including "User name", "Material ID", "Material name" and "Date".

If you select the Material ID such as "Mat_Astro", the window of "Login for Material" will appear. Enter the Password, and then the window of "Update and delete material data of Mat_Astro" appears.

4-4-4. The window of "Gel method list" including "User name", "Method ID", "Gel method name" and "Date".

If you select the Method ID such as "Standard_2DE", the window of "Login for gel method " will appear. Enter the Password, and then the window of "Update and delete gel method data of Standard_2DE" appears.

4-4-5. The window of "Analysis method list" including "User name", "Analysis ID", "Analysis method name" and "Date".

If you select the Method ID such as "Toda_org", the window of "Login for analysis method" will appear. Enter the Password, and then the window of "Update or delete analysis method data" appears.

4-5. Change user password

When you need to change "User Password", enter "User name" and "Password", and click on "Enter user name & password" button (Fig.57). Then the window of "Enter new password" appears. In this window, enter "New password" in each box.

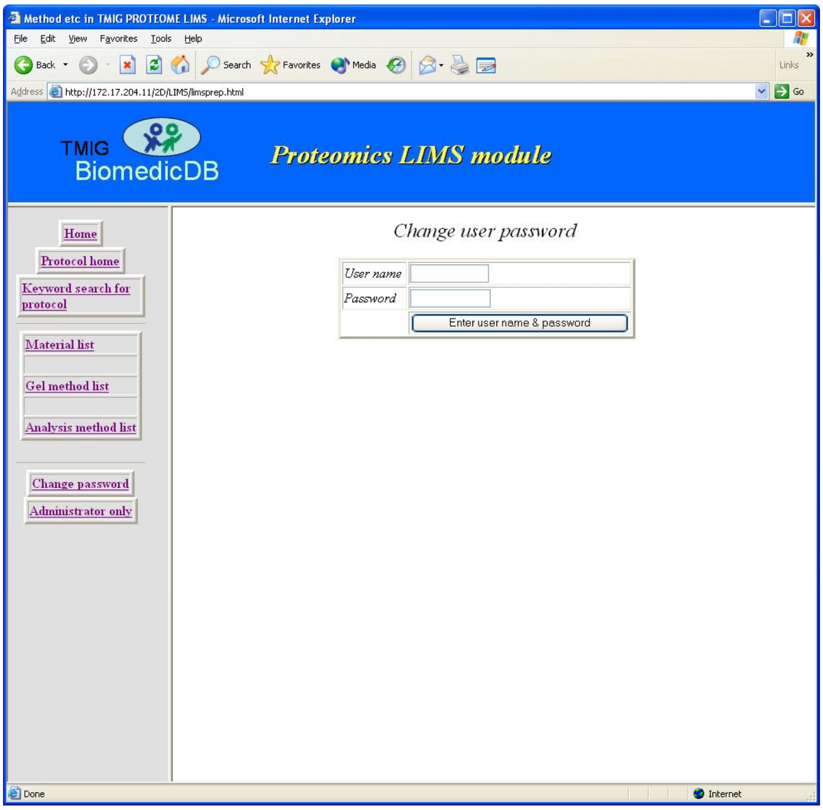

Fig.57

4-6. Administrator only

Only the administrator is allowed to use the window of "Add or delete user"(Fig.58). A new user is also able to be registered on this window (Fig.59).

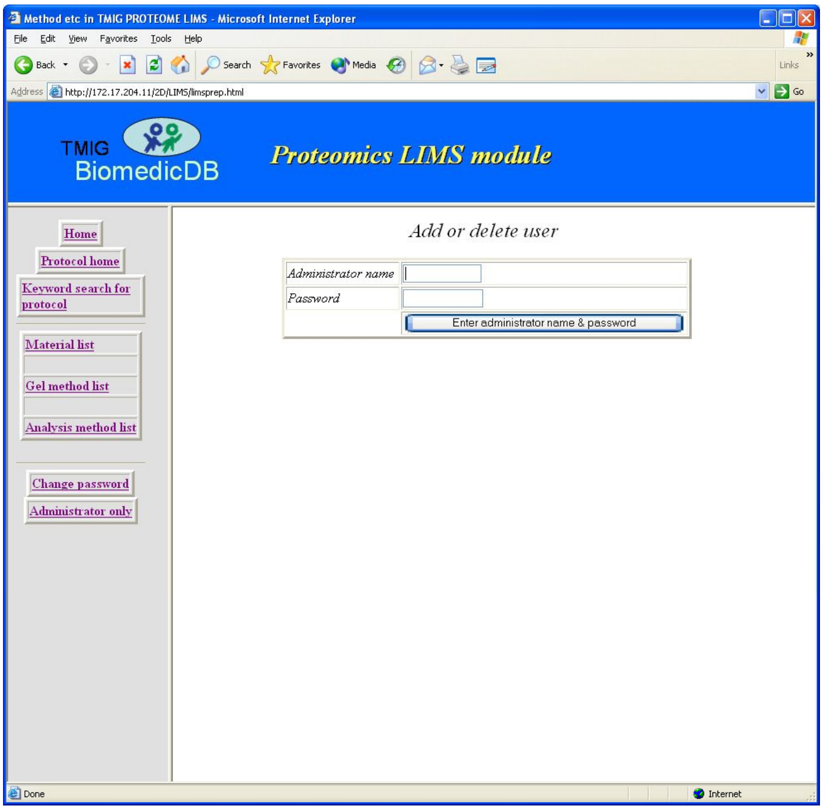

Fig.58

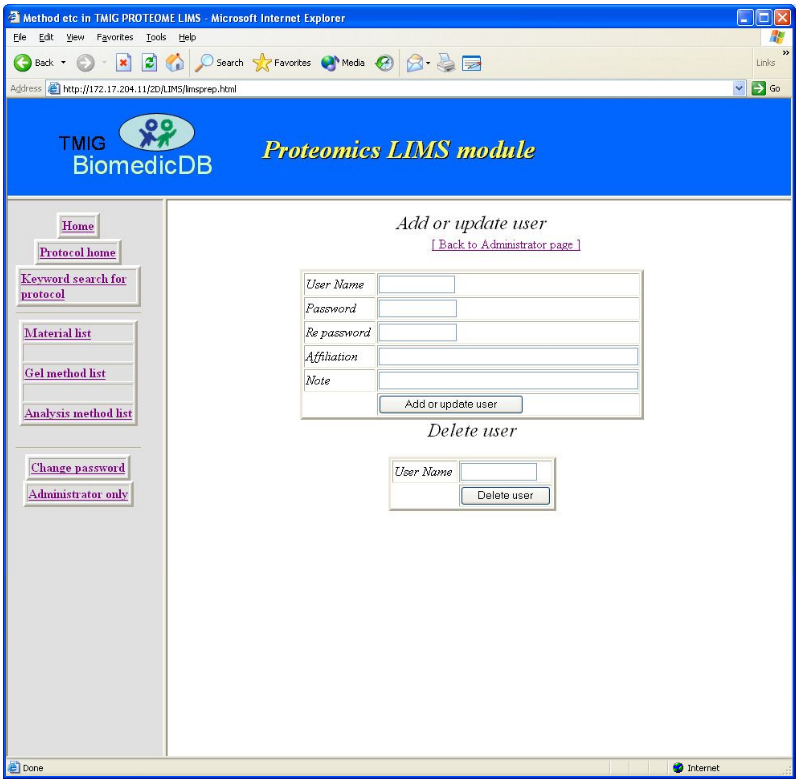

Method etc in TMIG PROTEOME LIMS - Microsoft Internet Explorer
http://172.17.204.11/2D/LIMS/limsprep.html
TMIG
BiomedicDB
Proteomics LIMS module
Home
Protocol home
Keyword search for protocol
Material list
Gel method list
Analysis method list
Change password
Administrator only
Add or update user
[Back to Administrator page]
User Name
Password
Re password
Affiliation
Note
Add or update user
Delete user
User Name
Delete user
Done
Internet

Fig.59

Chapter 5.　Installation

5-1　Setup

Software requirements : Apache revision 1.3.34 or later, PostgreSQL revision 7.4.3 or later, PHP revision 4.3.7 or later, GD library revision 2.0.27 or later. Please install them in advance.

And please set up as follows.

Please edit /usr/local/lib/php.ini

```
register_globals = On
```

```
#tar xvzf LIPAGE_****.tar.gz
#mv LIMS /usr/local/apache/htdocs
#cd /usr/local/apache/htdocs/LIMS
#su postgres
$createdb proteomedb
```

Please create and edit the first line in limsuser.back.

admin ID [tab] password [tab] affiliation [tab] occupational description [tab] admin(Input as a word)

[Using an example of user data:

```
#cp limsuser.euc limsuser.back
```

Usernames of the LIMS are toda and hmorisawa. The passwords are bmc27info and bmc36info.]

```
$psql -f createlimstable.sql proteomedb
```

The top page of the LIMS is http://servername/LIMS/lims.html.

5-2　Apache-PHP-PostgreSQL installation instruction using source files

The following description is applicable to Fedora Core 5.

Please install rpm-packages of gcc and enviroments for developments, first.
Please download each source files of apache_1.3.35.tar.gz, php-5.1.2.tar.gz, gd-2.0.33.tar.gz, jpegsrc.v6b.tar.gz,
libpng-1.2.6rc5.tar.gz and postgresql-8.1.3.tar.gz from the internet.

The download sites in the Internet

http://www.apache.org/
http://www.php.net/
http://www.boutell.com/gd/
ftp://ftp.uu.net/graphics/jpeg/
http://www.libpng.org/pub/png/libpng.html
http://www.postgresql.org/

[Apache installation]

```
#tar xvzf apache_1.3.35.tar.gz
#cd apache_1.3.35
#OPTIM="-O2" ./configure --enable-module=so
#make
#make install
#vi /usr/local/apahche/conf/httpd.conf

    ServerName    *********                    ( Enter a right name of server )

#vi /etc/rc.d/rc.local

   /usr/local/apache/bin/apachectl start       ( The line is added )
```

[PostgreSQL installation]

```
#adduser postgres
#passwd postgres
#mkdir /usr/local/src/postgresql-8.1.3
#chown postgres /usr/local/src/postgresql-8.1.3
#mkdir /usr/local/pgsql
#chown postgres /usr/local/pgsql
#su -postgres
$cd /usr/local/src
$tar xvfz postgresql-8.1.3.tar.gz
$cd /usr/local/src/postgresql-8.1.3
$./configure
$make
$make install

$vi /home/postgres/.bashrc

  PATH="$PATH":/usr/local/pgsql/bin
  export POSTGRES_HOME=/usr/local/pgsql
  export PGLIB=$POSTGRES_HOME/lib
  export PGDATA=$POSTGRES_HOME/data
  export MANPATH="$MANPATH":$POSTGRES_HOME/man
  export LD_LIBRARY_PATH="$LD_LIBRARY_PATH":"PGLIB"

$source ~/.bashrc
$initdb
$ cd /usr/local/pgsql/data
$chmod 600 pg_hba.conf
$vi pg_hba.conf
   #host    all    all    ::1/128    ident sameuser    ( Add #)
   host     all    0.0.0.0   0.0.0.0   trust            ( The line is added )
```

```
$su -
#vi /etc/rc.d/rc.local

    rm /tmp/.s.PGSQL.*                          ( The line is added )
    su - postgres -c "postmaster -S -i"         ( The line is added )
```

[zlib installation]

```
#tar xvzf zlib-1.2.1.tar.gz
#cd zlib-1.2.2
#./configure --shared
#make
#make install
```

[JPEG-6b installation]

```
#tar xvzf jpegsrc.v6b.tar.gz
#cd src/jpeg-6b
#./configure --enable-shared --enable-static
#make
#make install
```

[libpng installation]

```
#tar zxf libpng-1.2.6rc5.tar.gz
#cd libpng-1.2.6rc5
#cp scripts/makefile.linux makefile
#vi makefile

    ZLIBLIB=/usr/local/lib              ( The line is added )
    ZLIBINC=/usr/local/include          ( The line is added )
    #ZLIBLIB=../zlib                    ( Add #)
    #ZLIBINC=../zlib                    ( Add #)

#make
#make install
```

[GD installation]

```
#tar xvzf gd-2.0.28.tar.gz
#cd gd-2.0.28
#./configure
#make
#make install
```

[PHP installation]

```
#tar xvzf php-5.1.2.tar.gz
#cd php-5.1.2./configure --with-pgsql --with-apxs=/usr/local/apache/bin/apxs --enable-mbstr-enc-trans --enable-mbstring --enable-mbregex --enable-versioning --with-zlib-dir=/usr/local/lib --with-gd --with-jpeg-dir=/usr/local/lib
#make
#make install
```

```
#cp php.ini-dist /usr/local/lib/php.ini
#vi /usr/local/apache/conf/mime.types

     application/x-httpd-php    php                          ( The line is added )
     application/x-httpd-source    phps                      ( The line is added )

#cd ext/pgsql
#phpize
#aclocal
#./configure
#make
#make EXTENSION_DIR=/usr/local/lib/php/extensions install
#vi /usr/local/lib/php.ini

    extension_dir = /usr/local/lib/php/extensions        ( <-   extension_dir = ./   )
    extension        = pgsql.so                          ( The line is added )
    register_globals = On                                ( The line is added )

    ------------

#tar xvzf LIPAGE_****.tar.gz
#mv LIMS /usr/local/apache/htdocs
#cd /usr/local/apache/htdocs/LIMS
#cp limsuser.euc limsuser.back
#su postgres
$createdb proteomedb
$createuser nobody
```

[Using an example of user data:
   #cp limsuser.euc limsuser.back
   Usernames of the LIMS are toda and hmorisawa. The passwords are bmc27info and bmc36info.]

```
$psql -f createlimstable.sql proteomedb
```

The setup finished. The top page of the LIMS is http://servername/LIMS/lims.html.

5-3 Apache-PHP-PostgreSQL installations instruction using rpm packages on Fedora Core 4 and 5

The following description is applicable to Fedora Core 4 and 5.
Please select workstation install in installation type on Fedora Core 4 or 5

Please set up as follows.

```
#yum -y install httpd php php-gd php-pgsql
#yum -y install postgresql postgresql-server
#chkconfig httpd on
#chkconfig postgresql on
```

Please configure the three following files.

```
vi /etc/httpd/conf/httpd.conf
   User nobody          (<- User apache  )
   Group nobody         (<- Group apache  )

vi /etc/php.ini
   register_globals = On     (<- register_globals = Off)

vi /var/lib/pgsql/data/pg_hba.conf
   #host   all   all   ::1/128       ident sameuser      ( Add # )
   host    all   all   0.0.0.0   0.0.0.0   trust         ( The line is added )
```

Please reboot.

Please set up as follows.

```
#tar xvzf LIPAGE_****.tar.gz
#mv LIMS /var/www/html
#cd /var/www/html/LIMS
#cp limsuser.euc limsuser.back
#su postgres
$createdb proteomedb
$createuser nobody
```

[Using an example of user data:
#cp limsuser.euc limsuser.back
Usernames of the LIMS are toda and hmorisawa. The passwords are bmc27info and bmc36info.]

```
$psql -f createlimstable.sql proteomedb
```

The setup finished. The top page of the LIMS is http://servername/LIMS/lims.html.
